# Assessing Anosognosia in Apraxia of Common Tool-Use With the VATA-NAT

**DOI:** 10.3389/fnhum.2018.00119

**Published:** 2018-03-27

**Authors:** Ilka Buchmann, Rebecca Jung, Joachim Liepert, Jennifer Randerath

**Affiliations:** ^1^Department of Psychology, University of Konstanz, Konstanz, Germany; ^2^Lurija Institute for Rehabilitation and Health Sciences at the University of Konstanz, Schmieder Foundation for Sciences and Research, Allensbach, Germany; ^3^Kliniken Schmieder, Allensbach, Germany

**Keywords:** anosognosia, tool-use apraxia, VATA-NAT, psychometric properties, stroke

## Abstract

In neurological patients, a lack of insight into their impairments can lead to possibly dangerous situations and non-compliance in rehabilitation therapy with worse rehabilitation outcomes as a result. This so called anosognosia is a multifaceted syndrome that can occur after brain damage affecting different neurological or cognitive functions. To our knowledge no study has investigated anosognosia for apraxia of common tool-use (CTU) so far. CTU-apraxia is a disorder frequently occurring after stroke that affects the use of familiar objects. Here, we introduce a new questionnaire to diagnose anosognosia for CTU-apraxia, the Visual Analogue Test assessing Anosognosia for Naturalistic Action Tasks (VATA-NAT). This assessment is adapted from a series of VATA-questionnaires that evaluate insight into motor (VATA-M) or language (VATA-L) impairment and take known challenges such as aphasia into account. Fifty one subacute stroke patients with left (LBD) or right (RBD) brain damage were investigated including patients with and without CTU-apraxia. Patients were assessed with the VATA-L, -M and -NAT before and after applying a diagnostics session for each function. Interrater reliability, composite reliability as well as convergent and divergent validity were evaluated for the VATA-NAT. Seven percent of the LBD patients with CTU-apraxia demonstrated anosognosia. After tool-use diagnostics this number increased to 20 percent. For the VATA-NAT, psychometric data revealed high interrater-reliability (*τ* ≥ 0.828), composite reliability (CR ≥ 0.809) and convergent validity (*τ* = −0.626). When assessing patients with severe aphasia, the possible influence of language comprehension difficulties needs to be taken into account for interpretation. Overall, close monitoring of anosognosia over the course of rehabilitation is recommended. With the VATA-NAT we hereby provide a novel assessment for anosognosia in patients with CTU-apraxia. For diagnosing anosognosia we recommend to combine this new tool with the existing VATA-M and -L subtests, particularly in patients who demonstrate severe functional deficits.

## Introduction

Anosognosia (*α* = without, *νóσoς* = disease, *γνώσις* = knowledge) was first described by Babinski (1914; see Langer and Levine, 2014) as the “denial of motor deficits contralateral to a brain lesion” (Canzano et al., 2014). In the last few years the term has been used in a broader manner and has been extended to different clinical manifestations (Jenkinson and Fotopoulou, 2014; Turnbull et al., 2014). For example, Prigatano (2010) described anosognosia as “a multifaceted syndrome where patients show complete or partial lack of awareness of specific neurological or cognitive deficits”. It was shown for patients with cortical blindness (von Monakow, 1885; Anton, 1893), hemiplegia (Anton, 1893; Pia et al., 2004; Cocchini et al., 2010a; Vocat et al., 2010), aphasia (Lebrun, 1987; Rubens and Garrett, 1991; Cocchini et al., 2010b; Kertesz, 2010), hemianopia (Celesia et al., 1997) and dementia (Schacter, 1991; Starkstein et al., 2006; Orfei et al., 2010b; Spalletta et al., 2012; Cotelli et al., 2014). Anosognosia can occur after stroke, traumatic brain injury, neurodegenerative or neuropsychiatric disorders (Orfei et al., 2008; Prigatano, 2010). Patients behave as if they have no neurological or cognitive deficits or deliver evasive answers, why they cannot perform the requested action if they cannot execute a given activity (e.g., “The arm is tired”; Karnath, 2012). A distinction has been made between explicit and implicit anosognosia. If questioned about their abilities, patients with anosognosia, deny or underestimate their difficulties. Patients with explicit denial of the deficit may communicate that all is fine, yet they may avoid executing tasks that are difficult for them. In contrast, patients with implicit anosognosia tend to approach tasks although they cannot meet the demands and are not able to correct their behavior (Cocchini et al., 2010a; Garbarini et al., 2013). The complex picture of the disorder is complemented by the fact that selective anosognosia can occur, i.e., patients demonstrate no insight in one deficit but have good awareness for other deficits (Bisiach et al., 1986; Breier et al., 1995; Gasquoine, 2016). Further, it is striking that patients correctly identify errors made by others but are not able to recognize their own errors (Canzano et al., 2016).

Despite the fact, that patients often spontaneously recover from anosognosia symptoms (Starkstein et al., 2010; Vocat et al., 2010), it is a huge obstacle for a good therapy and rehabilitation process and predicts worse therapy outcome (Pedersen et al., 1996; Hartman-Maeir et al., 2001; Prigatano, 2008; Mattioli et al., 2012). Patients who do not recognize their deficits, typically show little therapy motivation and set unrealistic high goals for treatment (Fleming et al., 1998; Peskine and Azouvi, 2007). Further, they frequently reject assistance and treatment recommendations (Katz and Segal, 2004) and develop less compensatory strategies than patients without anosognosia (Ownsworth and McFarland, 2004).

Despite the increased interest in anosognosia over the past years to our knowledge no study so far specifically set out to investigate the possible existence of anosognosia for the impaired use of actual common tools and objects (common tool-use apraxia, here: CTU-apraxia). CTU-apraxia is a complex but yet underestimated dysfunction after lesions of the left hemisphere affecting tool and object use (Dovern et al., 2011; Goldenberg, 2011; Buchmann and Randerath, 2017). Patients for example may choose the wrong tool to perform an action (e.g., the toothbrush to comb their hair) or do not know the proper function of an object. Patients with apraxia are more often dependent on nursing staff (Poeck, 2006; Wu et al., 2014), its severity negatively predicts rehabilitation outcome (Hanna-Pladdy et al., 2003; Dovern et al., 2011) and patients with apraxia return less often to work than non apraxic patients (Dovern et al., 2011; Wang et al., 2014). Furthermore, Goldenberg (2011) described, that patients with tool-use apraxia may not realize their limitations or blame them on their aphasic or hemiplegic symptoms, and so do their relatives. However, studies on the topic of anosognosia in apraxia are scarce. To date there is one published study that examined anosognosia for bucco-facial apraxic deficits (Canzano et al., 2014).

But to our knowledge to date there is no published diagnostic instrument to assess anosognosia for CTU-apraxia. There exist some wide-ranging questionnaires for judging competencies of daily life activities, motor abilities, cognitive and emotional behavior (e.g., the Awareness Questionnaire by Hart et al., 2004; the Patient Competency Rating Scale, see Leathem et al., 1998; or the Marcel-Moro’s interview, Moro et al., 2011), which ask for activities of daily living and partly include self-ratings or clinical assessment but are neither clearly designed to evaluate apraxia nor to assess the insight into the impairment in CTU.

Here, we present the development of a new questionnaire to diagnose explicit anosognosia for CTU-apraxia, the Visual-Analogue Test assessing Anosognosia for Naturalistic Action Tasks (VATA-NAT).

To figure out whether participating in an assessment for limb apraxia Diagnostic Instrument for Limb Apraxia—Short Version (DILA-S; Buchmann and Randerath, 2017) may change the insight of the patients into their disabilities, the VATA-NAT was presented before and after carrying out the DILA-S which includes two tests with common tools (Familiar Tools Test (FTT) and NAT Breakfast Task).

Psychometric data will be analyzed with respect to interrater-reliability, internal consistency, convergent and divergent validity.

## Materials and Methods

### Participants

Participants were recruited from the neurorehabilitation clinic “Kliniken Schmieder” in Allensbach, Germany. The patients all suffered from first time stroke, were in the subacute or chronic phase of illness, did not require intensive care, could participate actively in therapy sessions and were resilient during 30 min of therapy. A total of 58 right handed stroke patients with left (LBD) or right brain damage due to stroke (RBD) participated. None of the patients suffered from any other neurological or psychiatric disease.

Due to false answer to the anosognosia control question (“Do you have any difficulties to jump over a lorry?”; see “Structure of the VATA-NAT” section), seven patients were excluded from further analysis. The remaining 51 patients were divided into four participant groups: LBD-patients with CTU-apraxia (LBD-A_CTU_, *n* = 15), LBD-patients without CTU-apraxia (LBD-nA_CTU_, *n* = 13), RBD-patients with CTU-apraxia (RBD-A_CTU_, *n* = 5) and RBD-patients without CTU-apraxia (RBD-nA_CTU_, *n* = 18). CTU-apraxia was assessed with the FTT and Naturalistic Action Test—Breakfast Task (NAT) of the DILA-S (Buchmann and Randerath, 2017) described below (see “Assessment of CTU-Apraxia” section).

Patient groups did not differ in sex, age, days since stroke onset and education level (*χ*^2^ ≤ 4.067, *p* ≥ 0.248). LBD patients with CTU-apraxia had worse speech comprehension than the other three groups (*U* ≥ 11.50, *p* ≤ 0.020). Lesion distribution (middle cerebral artery, brain stem, thalamus, pons, basal ganglia) appeared similar in the four patient groups. For demographic data see Table 1.

**Table 1:** Demographic and clinic data.

Group	LBD-A_CTU_ *n* = 15	LBD-nA_CTU_ *n* = 13	RBD-A_CTU_ *n* = 5	RBD-nA_CTU_ *n* = 18
Gender: male/female	8/7	6/7	2/3	8/10
Age	57.53 (30–78)	56.46 (41–79)	53.00 (27–78)	60.06 (25–79)
Days since stroke	60.80 (21–149)	103.00 (28–644)	68.00 (47–102)	55.28 (23–164)
Education level: middle/high	12/3	7/6	3/2	15/3
Receptive aphasia:				
No	4	9	4	18
Yes (mild/moderate/severe)	11 (5/2/4)	4 (3/1/0)	1 (1/0/0)	0
Lesion distribution:				
Middle cerebral artery	10 (66.7%)	8 (61.5%)	2 (40.0%)	16 (88.9%)
Brain stem	1 (6.7%)	0	0	0
Thalamus	1 (6.7%)	2 (15.4%)	0	0
Pons	0	0	0	1 (5.6%)
Basal ganglia	3 (20.0%)	3 (23.1%)	3 (60.0%)	1 (5.6%)

The study design was approved by the ethical committee of the University of Konstanz (Statement No. 10/2014). All patients were taking part in the study voluntarily. Informed consent was obtained, and privacy rights were observed. The study was conducted in accordance with the Declaration of Helsinki.

### Assessment of CTU-Apraxia

CTU-apraxia was assessed with two subtests of the DILA-S (Buchmann and Randerath, 2017): the FTT and the Naturalistic Action Test—Breakfast Task (NAT)^1^.

The FTT consists of three example items and five test items, each of them consisting of one object that was presented centrally on the table and three tools lying next to each other in front of the object. The participants were required to choose the correct tool to handle the recipient object. For example, one FTT item includes a pan with a fried egg inside as recipient object and three tools to choose from: a spatula, a bottle opener and a ladle. The patient was asked to choose the correct tool (e.g., the spatula) and to perform its typical application on the given recipient object (e.g., take the fried egg out of the pan). To avoid any influences of hemiplegia, all tasks were presented in a way that they could be managed unimanually with the ipsilesional hand. To classify patients as apraxic or not apraxic in simple CTU, we used the so-called Production Score. With this score the performance per executed action is evaluated in detail by judging grip-formation (for the fried egg example: lateral or tight cylinder grip), grip-orientation (e.g., thumb directed towards the functional part of the spatula), movement-content (e.g., move spatula towards the fried egg, slide it under the fried egg and take the fried egg out of the pan) and movement-orientation (e.g., upwards when taking the fried egg out of the pan). The cut-off value and maximum score consists of 20 points (based on norm-data acquired in 82 healthy subjects).

Additionally, patients were asked to perform a multi-step naturalistic action-task (NAT). Patients had to prepare one toasted slice of bread with butter and jam as well as a cup of tea with sugar. The cut-off score is set at four points, a maximum of six points can be achieved.

For a further description of these two tests please see Buchmann and Randerath (2017).

### Assessment of Anosognosia

#### Test Construction

Since apraxia and aphasia often co-occur after left brain damage and the severity of apraxia is correlated with the severity of aphasia, language comprehension and production deficits need to be considered when constructing an anosognosia test for apraxia. To account for the expected difficulties with language production and comprehension particularly in patients with left brain damage, the structure of our questionnaire follows the standard anosognosia tests for motor and language impairment: Visual-Analogue Test assessing Anosognosia for Motor Impairment (VATA-M; Della Sala et al., 2009) and Visual-Analogue Test assessing Anosognosia for Language Impairment (VATA-L; Cocchini et al., 2010b), which were developed to include aphasic patients. These questionnaires take two modalities into account: Reading/hearing the question and seeing a picture which illustrates the context to help aphasic patients to understand the instructions.

The focus of the VATA-NAT is not set on the motor deficits of patients, but rather on the patients’ ability to understand the concepts of planning and executing different actions, which is emphasized in the verbal instruction: “You now will be asked, how well you currently can solve a series of actions. Every task will be shown on a picture. Additionally, the question is written above the picture. I will read out loud every question to you. Please tell me, how well you can solve the action at this moment. The questions are not about the mobility of your arm, but generally, how you are able to mentally plan and execute the actions. Below each picture there is an evaluation scale. Please show me your capacity on this scale ranging from 0 to 3. Zero means, that you have no problem at all to solve the requested action at this moment. One means minor difficulties and two means serious difficulties. Three means, that it is currently not possible for you to solve the requested action. You can show me your answer by pointing on the appropriate position on the scale. Let’s start with a practice item”.

#### Structure of the VATA-NAT

The VATA-NAT comprises one example item, 13 test items and one control item. The example and control items were obtained from Della Sala et al. (2009). The control question asks about an impossible movement (“Do you have any difficulty to jump over a lorry?”) to check for language comprehension, perseverations and compliance of the tested patient. Patients who provided an incorrect response to this question were excluded from further study analysis (see “Participants” section).

Each item contains a drawing of a daily life activity performed unimanually. To confirm that the displayed actions are interpreted correctly, all images were first presented to five healthy volunteers who described the action. As a consequence, one of the original drawings was modified and one drawing was removed from the set because one of the volunteers had difficulties understanding the meaning of the picture, there are two versions: one with actions solved with the left and the other version demonstrates actions solved with the right hand for LBD or RBD patients, respectively.

Nine questions are related to single step tasks (take a fried egg out of a pan, open a bottle, scoop soup, clean a chalk-board, tighten a screw, fill a flower pot with soil, hang out the laundry, eat soup, use the phone) and four questions represent multi-step actions (prepare a slice of toast, prepare a cup of filtered coffee, punch paper and order it, set the table). Thirty percent of the illustrated actions were actually tested as part of the DILA-S (3 single step actions and 1 multi-step action). Overlap between diagnostics of CTU and the related anosognosia test provides the advantage of direct reference for the experimenter and patient.

**Figure 1:**
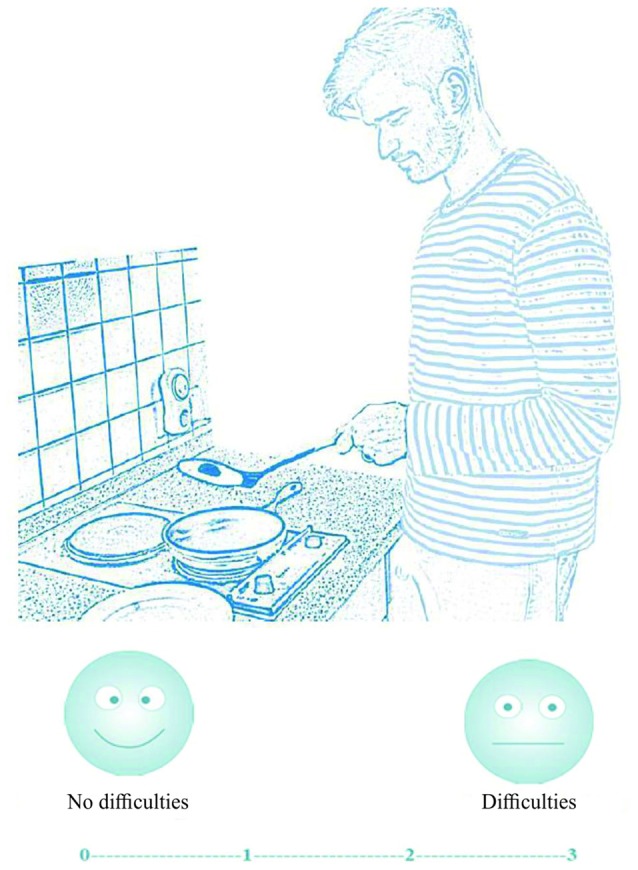
Illustration and question for the first single step item. Participants indicated their answer on the four point visual-analogue scale (0 = no difficulties, 1 = few difficulties, 2 = serious difficulties, 3 = impossible).

The items are presented in a fixed order with the single step action questions first, followed by the multi-step action questions and last the control question (for all questions see Supplementary Material). The items were represented on the patients’ ipsilesional site to reduce problems due to neglect or visuo-spatial deficits. Each question is presented on a sheet (Din A4) with one picture (or for multi-step actions with up to three pictures) and the visual-analogue answer scale underneath (see Figure 1). The experimenter read the question out aloud and explained the use of the visual-analogue answer scale. A score of 0 indicates “no difficulty in carrying out the task”, a score of 3 indicates “the task is impossible to carry out” (obtained from Della Sala et al., 2009). To help aphasic patients, two smileys indicate the respective ends of the scale: smiling (positioned above 0) and with a straight mouth (positioned above 3; see Figure 1).

#### Process and Evaluation of the VATA-NAT

The experimenter asks the patient to rate his/her actual ability to carry out the shown tasks despite his/her movement impairments. For each presented drawing the patient is asked in German to estimate his or her own ability to perform the shown activity such as: “Do you have any difficulty scooping soup?”. The patients indicate their answer on the 4-point visual-analogue scale. The control and the example items are not included in the final evaluation score for anosognosia.

The VATA questionnaires were administered twice. The mean time between the first and second measurement was *M* = 16 days (SD = 8 days, Range: 3–43 days). Patients rated their abilities twice, while the experimenter just rated the abilities of the patients once after CTU-apraxia tests.

To obtain a measure for anosognosia, the self-assessment outcome is compared with the evaluation of an independent rater. We deliberately did not choose to ask relatives, since these may be emotionally affected by the case and may not be familiar with the patients’ actual abilities (Ownsworth, 2014). Consequently, the experimenter rated the patients’ abilities in this study after the CTU-apraxia tests to obtain an objective reference value. The difference score between the patient and the experimenter score reflects the patients’ level of insight. The maximum score that can be reached for a judgment is 39 (13 items * 3 points). The patient’s self-rating was then subtracted from the experimenter’s rating. Accordingly, the potential range of the resulting experimenter-patient-discrepancy-value may vary between −39 to 39. A negative value indicates that the patient underestimated his abilities in reference to the experimenters’ opinion and a positive value indicates an overestimation (anosognosia).

#### Cut-Off Values for Interpretation

To better describe the grade of anosognosia for apraxic patients, cut-off values were determined. Since the variance of ratings between clinical raters may influence whether a patient may or may not breach conformity and thus may or may not be diagnosed with anosognosia, the cut-off value was deliberately not set at > 0. Instead, we here used the same approach applied by Della Sala et al. (2009) and Cocchini et al. (2010b) who determined the cut-off value for anosognosia by using interrater differences. In the current study, for ten patients there were ratings by two experimenters. The average difference score of these two raters was *M* = −0.10, SD = 2.64. The cut-off value for anosognosia of CTU-apraxia was set at the mean interrater difference plus two standard deviations (5.18). Because difference scores just reach half or whole numbers, the cut-off score was rounded to 5. This procedure was used in order to minimize the influence of interrater differences.

To provide an estimation of the severity of patient misjudgment we further distinguished between mild, moderate and severe anosognosia for CTU-apraxia. Mild to moderate anosognosia was defined by adding one point difference per item to the cut-off value (5.5–13), moderate anosognosia was determined by adding two points difference per item (ranging from 13.5 to 26) and severe anosognosia was defined by difference scores larger than 26.5 (see also Table 2).

**Table 2:** Cut-off values for anosognosia of common tool-use (CTU)-apraxia.

	No anosognosia	Mild anosognosia	Moderate anosognosia	Severe anosognosia
Cut-off values	≤ 5	5.5–13	13.5–26	26.5–39

#### Assessment of VATA-L and VATA-M

To further evaluate the patients’ ability of estimating their own motor and language skills, the VATA-L (Cocchini et al., 2010b) and VATA-M (Della Sala et al., 2009) were applied. Structure, processing, evaluation and interpretation methods are similar to the ones used for the VATA-NAT.

### Neuropsychological Assessment

Motor and language skills were further examined by using an adapted version of the Wolf Motor Function Test (WMFT; Wolf et al., 2001) and the “Naming” and “Token” subtests of the Aachener Aphasia Test (AAT; Huber et al., 1983). The WMFT was used to test the function of the contralesional arm and hand with eight items. Patients were allocated to one of two motor groups: one with minor or no motor deficits (median points ≥4 in WMFT) and one with moderate to severe motor deficits (median points < 4 points in WMFT). This was done to allow for divergent validity analysis (please see “Statistical Analyses” section). The AAT “Token” Test was used to test language comprehension. The patients were required to understand the instructions of the Token Test, otherwise their test results were excluded from further analysis. The AAT “Naming” Test was used to test word and sentence production abilities. Further, these three tests are consulted by the rater to evaluate the VATA-L (Cocchini et al., 2010b) and VATA-M (Della Sala et al., 2009) and thereby contribute to assessing anosognosia for aphasia and hemiplegia.

Furthermore, the Star Cancellation and Line Bisection Test (Plummer et al., 2003) were used to test for neglect. For those patients who showed neglect symptoms, the VATA-booklets were moved towards their less affected visual hemifield and patients were asked to also provide their answer verbally. Please note, that only one LBD-patient showed neglect symptoms, and this patient had to be excluded due to providing an incorrect answer for the control question. All other patients affected by neglect were RBD-patients without aphasic symptoms. These patients did not have any difficulties providing verbal responses to the VATA-NAT questions read aloud by the experimenter.

### Statistical Analyses

All behavioral analyses were conducted in IBM SPSS Statistics 24. Because no variables were normally distributed (tested with Chi^2^, *p* = 0.000), non-parametric tests were applied. To determine differences between all four patient groups Kruskall-Wallis-Tests with *post hoc* Mann-Whitney-U-Tests were used.

For interrater reliability and convergent validity, correlation analyses were applied (Kendall’s Tau). Internal consistency was measured with a conventional version of congeneric reliability (Composite Reliability, CR; Cho, 2016, p. 664).

Divergent validity was evaluated especially with regard to the involvement of motor function aspects. For this, we concentrated on self-ratings of patients without CTU-apraxia (LBD-nA_CTU_, *n* = 13 and RBD-nA_CTU_, *n* = 18) who were assigned to groups with moderate to severe motor deficits (LBD-nA_CTU_ *n* = 7; RBD-nA_CTU_ *n* = 12) and groups without or with only minor motor impairments (LBD-nA_CTU_ *n* = 6; RBD-nA_CTU_ *n* = 6). A group comparison and Kendall’s tau correlations of patients with or without motor difficulties were run separately for the subgroups of LBD and RBD patients, respectively, in order to assess whether motor function may have been considered in both patient self-ratings, in VATA-M but also in VATA-NAT. The reasoning is as follows: if self-ratings of patients without deficits in language comprehension or praxis (RBD-nA_CTU_) show differences according to the assigned motor function group, then motor function likely plays a role for judging the shown activities in the items (even though for the VATA-NAT it was instructed that the focus should be on action planning and even though actions were illustrated uni-manually). If instead these RBD-patients do not show difficulties to distinguish between VATA-NAT and VATA-M demands, but LBD-patients without motor and apraxic deficits do, it is likely that speech comprehension abilities play an important role in understanding the different VATA subtest demands.

## Results

### Patient Data

#### CTU-Apraxia

13 LBD- and 18 RBD-Patients did neither show any apraxic behavior in the FTT nor in the NAT and were therefore classified as patients without CTU-apraxia (LBD-nA_CTU_ and RBD-nA_CTU_).

15 LBD-Patients did show apraxic deficits in either the FTT or NAT or both. Six of these patients (40.0%) showed difficulties in the FTT Production Score, four (26.7%) in the NAT Breakfast Task and five patients (33.3%) showed difficulties with both tasks. These patients were assigned to the LBD-A_CTU_ group. In the RBD-patient-group five patients showed difficulties in either the FTT or NAT or both. Two RBD patients (40.0%) showed selective deficits in the FTT Production Score, two patients (40.0%) only demonstrated deficits in the NAT Breakfast Task and one patient (20.0%) showed difficulties in both tasks. These patients were assigned to the RBD-A_CTU_ group.

#### Anosognosia of Limb Apraxia

##### Patients with CTU-apraxia

**Figure 2:**
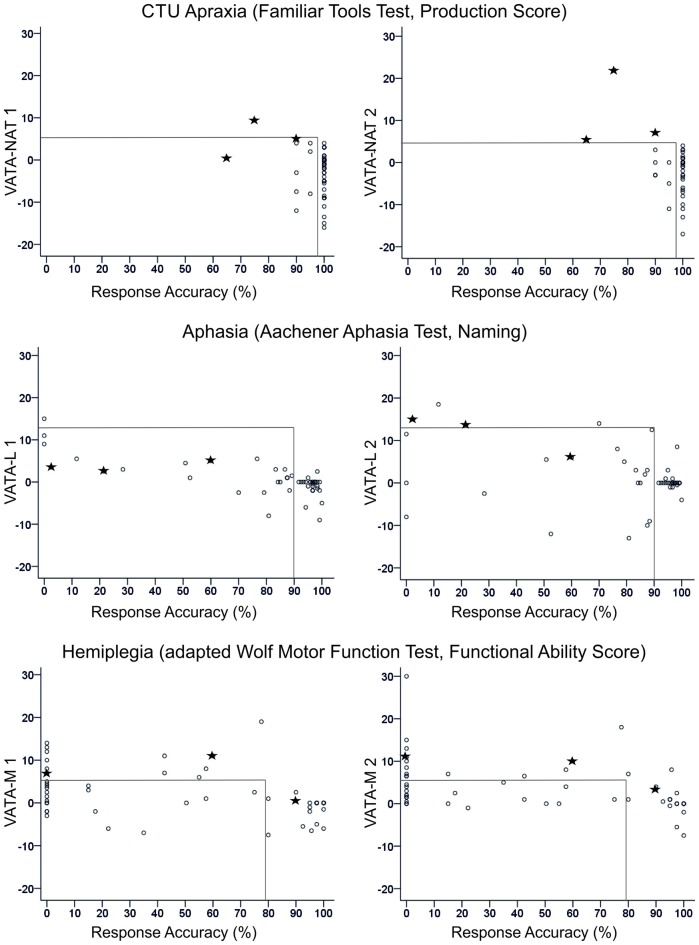
Overview of all patients’ difference scores in Visual-Analogue Test assessingAnosognosia for Naturalistic Action Tasks (VATA-NAT; above), Visual-Analogue Test assessing Anosognosia for Language Impairment (VATA-L) (center) and Visual-Analogue Test assessing Anosognosia for Motor Impairment (VATA-M) (below) at timepoint 1 (begin, left side) and 2 (end, right side). Cut-off values for the VATAs and the corresponding ability tests familiar tools test (FTT Production Score, Aachener Aphasia Test (AAT) Naming and Wolf Motor Function Test (WMFT) Functional Ability Score) are depicted with gray lines in the figures. The three patients who show anosognosia for common tool-use (CTU)-apraxia at timepoint 2 (LBD028, LBD53, LBD098) are consistently marked by black stars in all figures.

After testing CTU-apraxia, LBD patients with CTU-apraxia showed higher values in the VATA-NAT questionnaire as demonstrated in Table 3 and Figure 2. All three patients with anosognosia were diagnosed with severe apraxia in the NAT Breakfast Task. Two of them (LBD028, LBD098) also showed severe impairments in the FTT Production Score and one (LBD053) showed moderate disabilities. Six patients underestimated their abilities before being tested with the FTT and NAT Breakfast Task, four of these patients did this also after being tested.

**Table 3:** Frequencies of anosognosic patients in the LBD- and RBD-A_CTU_-groups.

	No anosognosia	Mild anosognosia	Moderate anosognosia	Severe anosognosia
Number of affected patients before testing CTU-apraxia in LBD-A_CTU_-group (*N* = 15)	14 (93%)	1 (7%)	0	0
Number of affected patients after testing CTU-apraxia in LBD-A_CTU_-group (*N* = 15)	12 (80%)	2 (13%)	1 (7%)	0
Number of affected patients before testing CTU-apraxia in RBD-A_CTU_-group (*N* = 5)	5 (100%)	0	0	0
Number of affected patients after testing CTU-apraxia in RBD-A_CTU_-group (*N* = 5)	5 (100%)	0	0	0

In RBD-A_CTU_-patient-group no patient showed anosognosia symptoms in neither the first nor the second time tested. This could be due to the mild CTU-apraxia symptoms. In FTT Production Score all patients reached at least 90% of performance and in NAT Breakfast Task only three out of five patients showed apraxic symptoms. Underestimation occurred in one patient before being tested with the FTT and NAT Breakfast Task, and two of the RBD-A_CTU_-patients underestimated their abilities after being tested.

##### Effects of testing timepoint and severity of impaired functions

Figure 2 delivers an overview of the distribution of all patients’ anosognosia scores in the VATA subtests for testing timepoint 1 (begin) and 2 (end) and their corresponding neuropsychological or motor test values indicating the degree of apraxia, aphasia or hemiplegia, respectively.

For all subtests, session 2 demonstrated a higher frequency of diagnosed anosognosia in the respective function of CTU, motor or language abilities. The severity of functional deficits was associated with an increased overestimation of ones’ own abilities therein (see Table 4).

**Table 4:** Relationships between anosognosia and the respective abilities.

Domain	Test	VATA Difference Score 1	VATA Difference Score 2
Apraxia	FTT production	*τ* = −0.174	*τ* = −0.255
(VATA-NAT)		*p* = 0.130	*p* = 0.031
Aphasia	AAT token	*τ* = 0.377	*τ* = 0.297
(VATA-L)		*p* = 0.001	*p* = 0.009
	AAT naming	*τ* = −0.468	*τ* = −0.253
		*p* = 0.000	*p* = 0.016
Motor function	WMFT	*τ* = −0.320	*τ* = −0.366
(VATA-M)		*p* = 0.002	*p* = 0.000

##### Specificity of anosognosia

**Figure 3:**
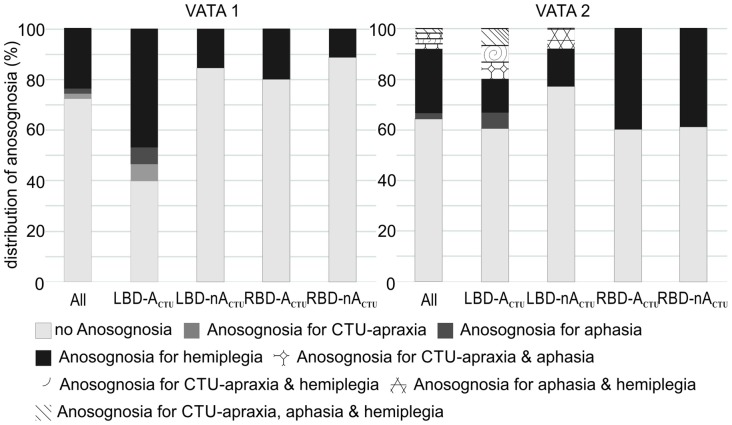
Distribution of anosognosia including overlapping incidence of CTU-apraxia, aphasia and hemiplegia for the entire sample (All, *N* = 51) and splitted per patient group (LBD patients with CTU-apraxia: LBD-A_CTU_ (*n* = 15); LBD patients without CTU-apraxia: LBD-nA_CTU_ (*n* = 13); RBD patients with CTU-apraxia: RBD-A_CTU_ (*n* = 5); RBD patients without CTU-apraxia: RBD-nA_CTU_ (*n* = 18)).

While in the first test session of VATA subtests no patient showed any overlap of anosognosia for the different neurological symptoms of CTU-apraxia, aphasia and hemiplegia, in the second assessment, a few individuals did (please see Figure 3). LBD028 overestimated her abilities in all three neurological symptoms. Further, three patients overestimated their abilities in two of the syndromes: LBD053 for CTU-apraxia and hemiplegia; LBD098 for CTU-apraxia and aphasia and LBD018 for aphasia and hemiplegia.

### Psychometric Data

#### Interrater-Reliability

Interrater-reliability was measured with data of 10 LBD-patients with (*n* = 6) or without (*n* = 4) CTU-apraxia by I.B. and A.M. In LBD-patients without CTU-apraxia interrater-reliability was very high (*τ* = 1.000, *p* = 0.000), but also for LBD-patients with CTU-apraxia high agreement was achieved (*τ* = 0.828, *p* = 0.022).

#### Internal Consistency

Internal consistency of the VATA-NAT questionnaire was examined separately for test and retest sessions for the patients’ evaluation across all groups. In addition, it was evaluated for the experimenter rating. Results indicated very good internal consistency for the VATA-NAT (patients’ 1st session: CR = 0.809; patients’ last session: CR = 0.845; experimenter: CR = 0.906).

#### Convergent Validity

Correlations between VATA-NAT and the apraxia scores revealed a strong association between the VATA-NAT experimenter score and the FTT (*τ* = −0.626, *p* = 0.003) in the LBD-A_CTU_-group. It was shown, that patients who performed better in the FTT were also evaluated to perform better in actions illustrated in the VATA-NAT questionnaire (corresponding to lower scores). There was only a weak correlation between the VATA-NAT experimenter score and performance in the NAT (*τ* = −0.319, *p* = 0.118) in the LBD-A_CTU_-group, whereby it needs to be noted that the overall range in NAT results is quite limited (0–6 points) and the NAT consists only of one complex sequential task.

For RBD-A_CTU_-patients, LBD- and RBD-patients without CTU-apraxia, no correlations could be carried out due to corresponding missing variance in data.

The VATA-L experimenter score correlated significantly with the “Token” and “Naming” subtests of the AAT for patients with receptive and productive aphasia, respectively (Token: *τ* = 0.542, *p* = 0.004; Naming: *τ* = −0.516, *p* = 0.001). Patients with more errors in the AAT “Token” Test and with less points for speech production were assessed worse in the VATA-L (corresponding to higher experimenter scores). Due to missing variance, no correlations were correlated for the patients without aphasia.

The VATA-M experimenter score correlated significantly with the WMFT scores for patients with motor impairments (*τ* = −0.466, *p* = 0.001). Patients with more difficulties in the WMFT were evaluated to perform worse in the actions illustrated by the VATA-M questionnaire (corresponding to higher experimenter ratings).

Correlations of VATA-NAT difference scores with days since stroke onset revealed no significant correlations (τ ≤ 0.600, *p* ≥ 0.058).

#### Divergent Validity

To test for the influence of motor skills on the evaluation of VATA-NAT questions, the RBD-nA_CTU_-group was divided into two subgroups, one with moderate to severe motor deficits (WMFT mean points < 4; *n* = 12) and one with minor or no motor deficits (WMFT mean points ≥ 4; *n* = 6). These patient groups did not differ in their evaluation of their daily living skills in VATA-NAT (begin: *U* = 34.00, *p* = 0.869; end: *U* = 34.00, *p* = 0.890). Also, the experimenter ratings of the patients’ abilities did not differ between groups (*U* = 33.50, *p* = 0.855).

To analyze the influence of possible language comprehension impairments going along with left brain damage, the LBD-nA_CTU_-group was analyzed in the same way. In the VATA-NAT, the patients’ self-evaluations differed between patients with (*n* = 7) or without (*n* = 6) motor difficulties (*U* ≥ 1.00, *p* = 0.003). This group-difference did not show for the experimenter evaluation (*U* = 10.00, *p* = 0.103).

At the same time, the VATA-M patient as well as experimenter scores both differed between patient groups with or without motor difficulties (*U* ≥ 0.00, *p* ≤ 0.011). Further, VATA-NAT patient scores of LBD-nA_CTU_ patients correlated with VATA-M patient scores and WMFT points (*τ* ≥ 0.532, *p* ≤ 0.024). Accordingly, patients with lower motor abilities in the WMFT or VATA-M self-evaluation also scored themselves to perform worse on actions illustrated in the VATA-NAT questionnaire (corresponding to higher VATA-NAT values). The experimenter rated both patient groups with or without motor disabilities on an equal level in planning and executing daily live activities (VATA-NAT; *U* = 10.00, *p* = 0.103). Please note that the LBD patients who demonstrated anosognosia for their motor abilities (begin: *n* = 2; end: *n* = 3) had worse comprehension abilities than RBD-patients (*U* = 72.00, *p* = 0.008) which could have influenced these results.

VATA-NAT experimenter scores did not correlate with the patients’ performance in the AAT “Token” test (*τ* = 0.416, *p* = 0.088).

## Discussion

Anosognosia, particularly for motor impairment, has been frequently observed going along with right brain damage. This predominance of patients with right brain damage in studies on anosognosia could be explained by the typically investigated subtype, namely anosognosia for hemiplegia, excluding patients with aphasia from these studies to ensure that difficulties with language will not affect the data (Morin, 2017). With the VATA-NAT we introduce a new questionnaire for explicit anosognosia of CTU-apraxia. CTU-apraxia is a typical impairment after left brain damage, frequently going along with language deficits. To be able to assess anosognosia also in patients with aphasia, the structure of the VATA-NAT was adapted to that of the VATA-M and VATA-L, two reliable and valid instruments, which measure anosognosia for hemiplegia and aphasia including patients with comprehension difficulties. For the VATA-subtests a patient’s self-rating and a clinical assessment is obtained, and the difference score is taken as measure for anosognosia. To circumvent the problem of subjectivity by relatives, the clinical evaluation of the VATA-NAT was carried out by experimenters. It has been shown before, that especially in the acute phase relatives may have little information about the actual state of the patient (Starkstein et al., 2006; Cocchini et al., 2010b) and that stress and anxiety can influence their evaluation of the patients’ abilities (Prigatano et al., 2005; Orfei et al., 2010a; Gambina et al., 2015).

Overall psychometric data showed that the tested VATA-NAT is a reliable and valid tool, demonstrating high levels of internal consistency and convergent validity. The experimenter rating revealed very good interrater reliability and enabled us to calculate cut-off values to estimate the degree of unawareness. The limitations found for divergent validity with respect to motor abilities will be discussed in more detail below.

### Incidence of Anosognosia of CTU-Apraxia in the Current Sample

In this study, it was shown that during the observed course of rehabilitation in a subacute patient sample 20 percent of LBD-patients with CTU-apraxia suffered from anosognosia (*n* = 3) with one patient showing moderate and the other two mild anosognosia. These three patients demonstrated moderate to severe CTU-apraxia.

In the RBD group only a few patients were mildly affected by CTU-apraxia and did not demonstrate any anosognosia. Thus, while several studies emphasized that anosognosia mainly follows right (Geschwind, 1965; Pedersen et al., 1996; Pia et al., 2004; Baier and Karnath, 2005; Baier et al., 2014) or bilateral brain damage (in 60% of 20 reviewed studies; Orfei et al., 2009), the current results are in line with other studies which have shown that anosognosia for motor (Cohen et al., 1991; Cocchini et al., 2009) or language difficulties (Kertesz and Benson, 1970; Maher et al., 1994; Cocchini et al., 2010b) after left brain damage is possible.

Further, patients who were more impaired in the FTT Production Score were more likely to show high difference scores in the VATA-NAT. In fact, for all VATA subtests, the severity of functional deficits was associated with an increased overestimation of ones’ own abilities therein. This is in line with previous studies which showed that aphasic patients with anosognosia showed lower performance scores in language tests (Cocchini et al., 2010b) and less self-correction behavior (Dean et al., 2016) than patients without anosognosia. Similarly, patients with anosognosia of dementia showed worse performance in autobiographic memory tasks (Berlingeri et al., 2015) and daily life activities (Gambina et al., 2015). The association of anosognosia with severe functional deficits is in line with the idea that a clear disturbance of intellect, memory and/or attention might be crucial for developing anosognosia (Karnath, 2012).

### Influence of Impaired Language and Motor Function

Our questionnaire was adapted from a series of VATA subtests that use drawings of activities representing the situation to be judged and a visual-analogue scale to indicate the judgment: VATA-L for language functions (Cocchini et al., 2010b) and VATA-M for motor functions (Della Sala et al., 2009). The items of the introduced VATA-NAT consist of drawings of naturalistic action tasks and were developed to evaluate CTU-apraxia patients with respect to their insight into their impairments in daily life activities involving objects. Anosognosia was indicated by the response deviation between the patient’s self-evaluated capabilities in the presented object interactions and the assessment of the experimenter using the same items. Unlike structured interviews, which often exclude aphasic patients from anosognosia screening (Cutting, 1978; Cocchini et al., 2009, 2012), the VATA subtests were designed to take language difficulties into account. Since CTU-apraxia severity highly correlates with aphasia severity, this is a very relevant feature. However, despite these undertaken measures language comprehension appears to still influence the patient’s response in the here introduced test as will be explained below.

A clear disadvantage is that particularly for patients with left brain damage a differentiation between the judgments based on motor difficulties and based on action planning cannot be clearly made for the VATA-NAT. Left brain damaged patients without CTU-apraxia demonstrated an influence of motor impairment in judging the capabilities for the displayed actions. Since this was not found for RBD-patients, it has to be concluded that aphasia is impeding the possibility to answer VATA-NAT questions according to the given instructions. We recommend to first of course pay attention to clearly instruct the VATA-NAT, but to additionally collect VATA-M data, which may deliver an additional insight into the patient’s self-evaluative behavior. Further, the additional collection of VATA-M data is important, since recent studies including the current showed that around 40% of LBD-patients demonstrate unawareness for their motor deficits, most of them moderately to severe (Della Sala et al., 2009; Nurmi and Jehkonen, 2014).

Thus, our data suggests that LBD-patients with CTU-apraxia may interpret the presented naturalistic actions in a more holistic way, i.e., including the evaluation of motor deficits next to apraxic impairment instead of following the instructions that put the focus on the evaluation of action planning capabilities only. This needs to be taken into account when applying this questionnaire.

### Effects of Timepoint

We measured anosognosia for motor and language impairment as well as anosognosia for deficits in CTU at two timepoints within the subacute rehabilitation phase. In between these testings, a diagnostic session took place assessing motor abilities, language comprehension and production as well as CTU performance. The performance in the diagnostic session was used as a reference for the clinical rater to evaluate the patient’s abilities.

It was striking that in all three VATA-tests (VATA-L, -M and -NAT) the frequency of diagnosed anosognosia increased at timepoint two. One likely reason for this is that patients may have taken their spontaneous or rehabilitation related recovery into account and judged their performance to be better at point two. Further, the therapists’ practices (e.g., positive feedback) to keep the patient’s motivation on a high level during training sessions may play an additional role.

Thus, to enhance sensitivity for potential misjudgments of performance especially during the subacute phase it is highly recommended to both monitor functional deficits and awareness in parallel over time. Future studies investigating anosognosia in a subacute phase need to consider these fast changes and should disentangle factors that influence anosognosia outcomes and may find measures for differentiating between an overly positive attitude vs. anosognosia. Further, our study shows impressively that confrontation with the fact that one’s action cannot be performed does not help to gather a better awareness of one’s disabilities. We propose that anosognosia should be taken into account when developing and applying rehabilitation approaches for patients with CTU-apraxia.

### Limitations and Recommendations

To our knowledge this was the first study which aimed to develop a questionnaire for anosognosia of CTU-apraxia. Based on a relatively small sample of 20 subacute patients with CTU-apraxia this study demonstrated that anosognosia can occur. With this study, a first step towards a reliable and validated questionnaire is taken. However, methods should be developed that may allow to improve divergent validity and better extract the motor component when assessing anosognosia for CTU-apraxia in left brain damaged patients with aphasia. Further, future studies should aim at including a higher number of patients with CTU-apraxia following them across different phases of rehabilitation (see for example: Vocat et al., 2010). Ideally for statistical comparisons patient groups should have the same size.

Further, we refrained from evaluating test-retest reliability for two reasons: first, to be consistent with our hypothesis that the confrontation with diagnostics may influence retest results and second, the fact that in a group of subacute patients spontaneous and rehabilitation related recovery should play a significant role. To be able to calculate test-retest reliability future studies should include a repeated testing with a short time interval (e.g., 24 h after the first session).

Since anosognosia is a crucial symptom influencing rehabilitation motivation and rehabilitation outcome, it is recommended to test for it. When using the here presented VATA-NAT, the influence of motor impairment should be taken into account. We recommend combining the different VATA subtests.

Future studies need to address the underlying mechanisms that may explain anosognosia for CTU-apraxia. One of several possible explanations to build up on is for example the idea that unawareness is at least in part a higher-order deficit of motor intention or planning, which is both an intrinsic component of the neglect syndrome’ as stated by Vallar et al. (2003, p. 297), as well as a core aspect of apraxia.

## Author Contributions

IB collected and analyzed most of the patient data. RJ developed the introduced test together with JR and collected part of the data. JL was the contact person for all clinical work, discussed the design and helped with writing the manuscript. JR had the idea for the here introduced research and wrote the manuscript together with IB.

## Conflict of Interest Statement

The authors declare that the research was conducted in the absence of any commercial or financial relationships that could be construed as a potential conflict of interest.

## Footnotes

^1^For materials and examples please see https://www.moco.uni-konstanz.de/publikationen/assessments/

## Abbreviations

AAT: Aachener Aphasia Test
CTU: Common tool-use
DILA-S: Diagnostic Instrument for Limb Apraxia—Short Version
FTT: Familiar Tools Test
LBD: Left brain damage due to stroke
NAT: Naturalistic Action Test
RBD: Right brain damage due to stroke
VATA-L: Visual-Analogue Test assessing Anosognosia for Language Impairment
VATA-M: Visual-Analogue Test assessing Anosognosia for Motor Impairment
VATA-NAT: Visual-Analogue Test assessing Anosognosia for Naturalistic Action Tasks
WMFT: Wolf Motor Function Test.
