# Supplementary material for: Assessing Anosognosia in Apraxia of Common Tool-Use With the VATA-NAT
Source: Front Hum Neurosci. 2018 Mar 27;12:119. doi: 10.3389/fnhum.2018.00119 (PMC5880953; doi:10.3389/fnhum.2018.00119)
Supplement: Supplementary file 1 [file Data_Sheet_1.PDF]

VATA-NAT Evaluation Sheet

Name: ______________ Date: ____________

No.	Task	Patient	Examiner	O	U	C
E.g..	*drive a car*		*Task understood?*			
1	take a fried egg out of a pan					
2	open a bottle					
3	scoop soup out of a pot					
4	clean the board					
5	tighten a screw					
6	fill a flower pot with potting soil					
7	hang out the laundry					
8	eat soup					
9	use the phone					
10	prepare a slice of toast					
11	prepare a cup of filter coffee					
12	punch paper and order it					
13	set the table					
**Sum**						
Check	*jump over a lorry*		*correct? (expected score: 2-3)*<br>☐ *yes* ☐ *no*			

O = Overestimation, U = Underestimation, C = correct estimation (Difference ≤ ±0.5)

Difference Score (Experimenter Score – Patient Score): ________
(**Attention**: this is calculated without example and control question)

Interpretation (**Attention**: this is not meaningful, if control question was answered incorrect)

**Disparity Score**	< -1	-1 to 5	5.5 to 13	13.5 to 26	> 26
**Interpretation**	Under-estimation	correct estimation	mild anosognosia	moderate anosognosia	severe anosognosia
