# Supplementary material for: Assessing Anosognosia in Apraxia of Common Tool-Use With the VATA-NAT
Source: Front Hum Neurosci. 2018 Mar 27;12:119. doi: 10.3389/fnhum.2018.00119 (PMC5880953; doi:10.3389/fnhum.2018.00119)
Supplement: Supplementary file 2 [file Data_Sheet_2.PDF]

Supplementary Material

Anosognosia in Apraxia of Common Tool-Use

Ilka Buchmann [1,2], Rebecca Jung [1], Joachim Liepert [2,3] & Jennifer Randerath [1,2*]

[1] University of Konstanz, Konstanz, Germany

[2] Lurija Institute for rehabilitation sciences and health research at the University of Konstanz, Konstanz, Germany

[3] Kliniken Schmieder, Allensbach, Germany

[*] **Correspondence:**

Dr. Jennifer Randerath,

Department of Psychology, University of Konstanz & Lurija Institute, Kliniken Schmieder Allensbach, Pb 216, Universität Konstanz, Universitätsstr. 10, 78457 Konstanz, Germany

email: J_Randerath@hotmail.com, jennifer.randerath@uni-konstanz.de

1 Supplementary Data

The VATA-NAT Booklet is provided separately for patients with left and right brain damage. Consequently, the actions executed on the images are shown with the left or right hand.

LURIJA INSTITUT

Universität Konstanz

Booklet for the Visual-Analogue Test assessing Anosognosia for Naturalistic Action Tasks

(VATA-NAT)

For Patients with left hemisphere brain damage

VATA-NAT

Instructions

"You now will be asked, how well you **currently** can solve a series of actions. Every task will be shown on a picture. Additionally, the question is written above the picture. I will read out every question to you. Please tell me, how well you can solve the action **at the moment**. The questions are **not** about the mobility of your arm, but generally, how you are able to mentally **plan and execute the actions**.

Below each picture there is an evaluation scale. Please show me your capacity on this scale ranging from 0 to 3. Zero means, that you have no problem at all to solve the requested action at this moment. One means minor difficulties and two means serious difficulties. Three means, that it is currently not possible for you to solve the requested action. You can show me your answer by pointing on the appropriate position on the scale. Let´s start with a practice item."

Example: Do you have any difficulties to drive a car?

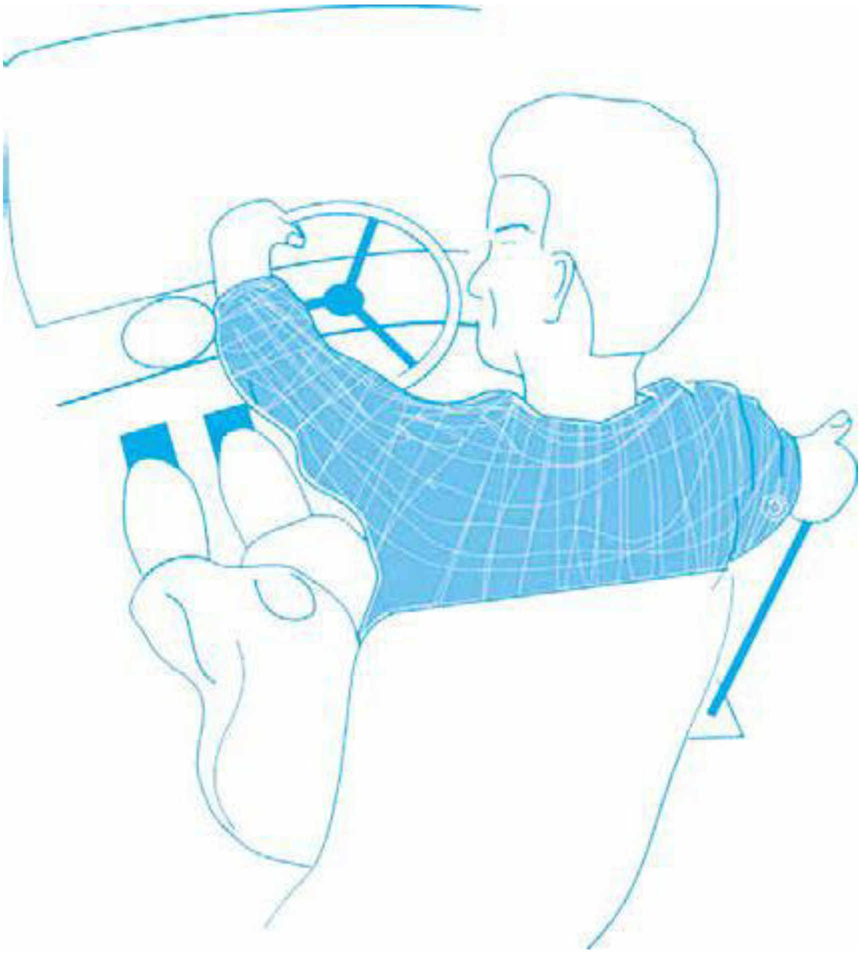

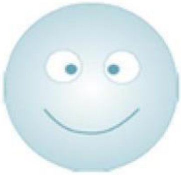

No difficulties

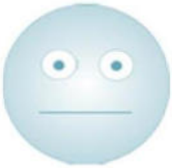

Difficulties

0------------------------1------------------------2------------------------3

1. **Do you have any difficulties to take a fried egg out of a pan?**

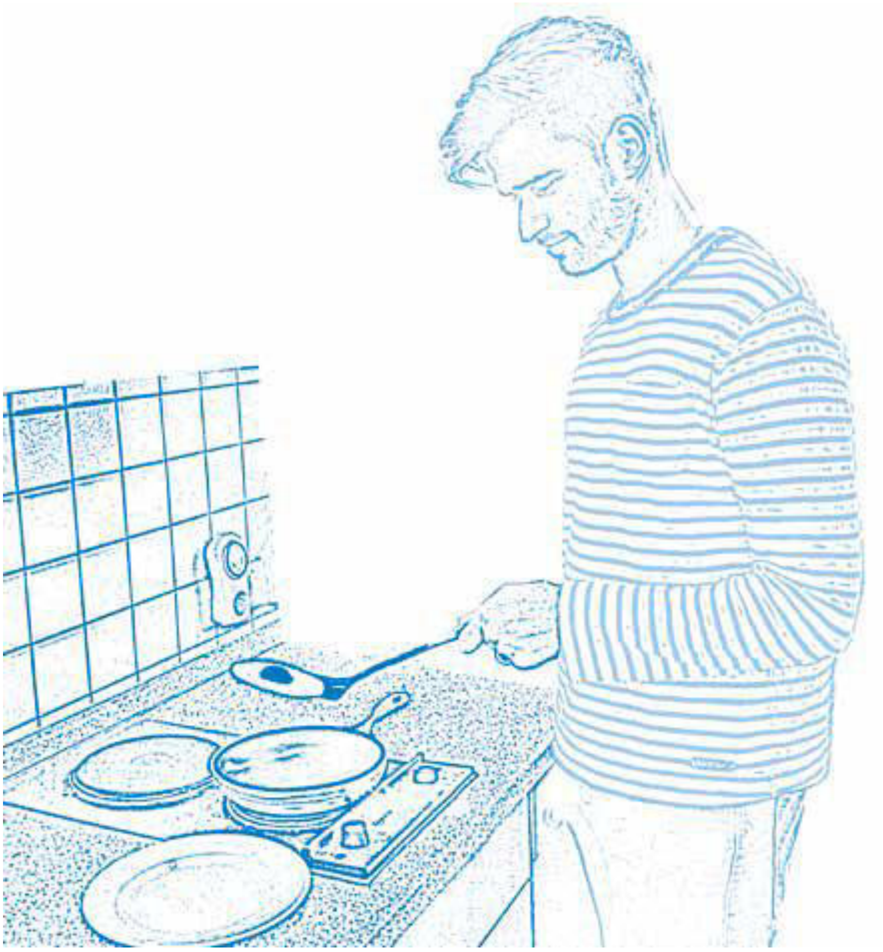

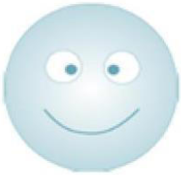

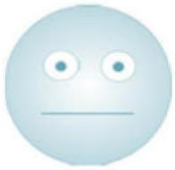

No difficulties **Difficulties**

0------------------------1------------------------2------------------------3

2. Do you have any difficulties to open a bottle with crown cap?

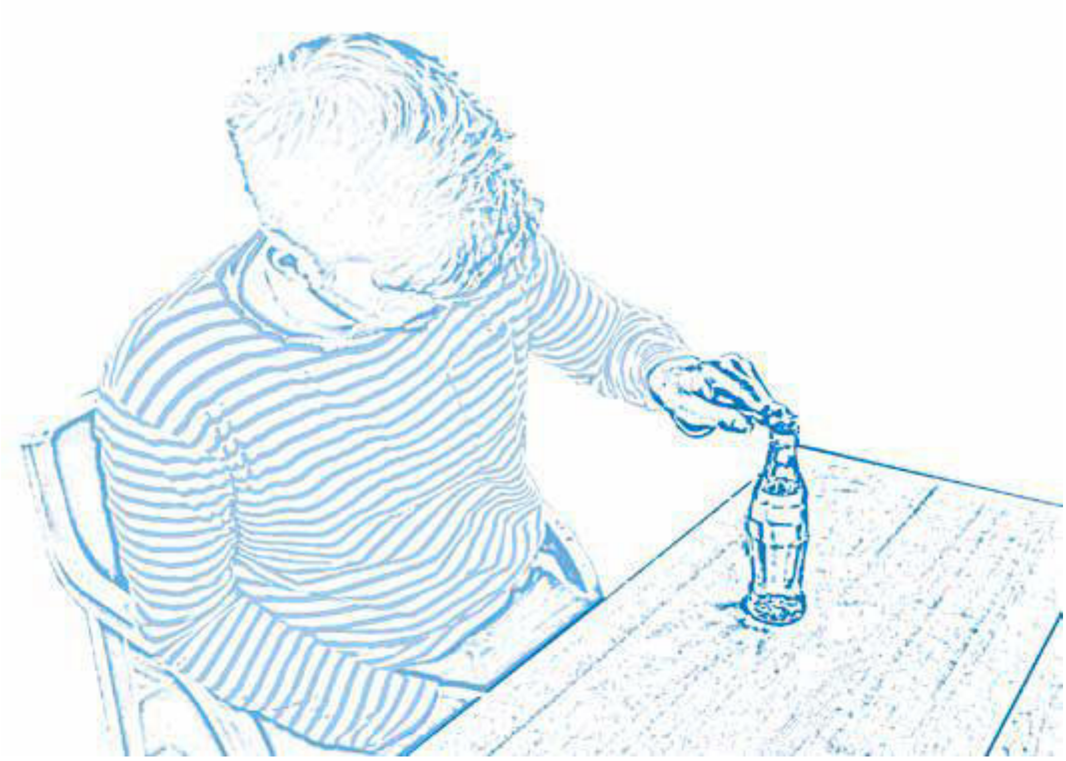

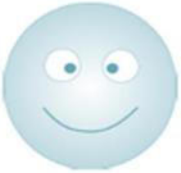

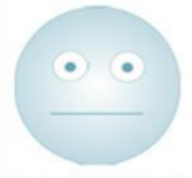

No difficulties **Difficulties**

0------------------------1------------------------2------------------------3

3. Do you have any difficulties to scoop soup out of a pot?

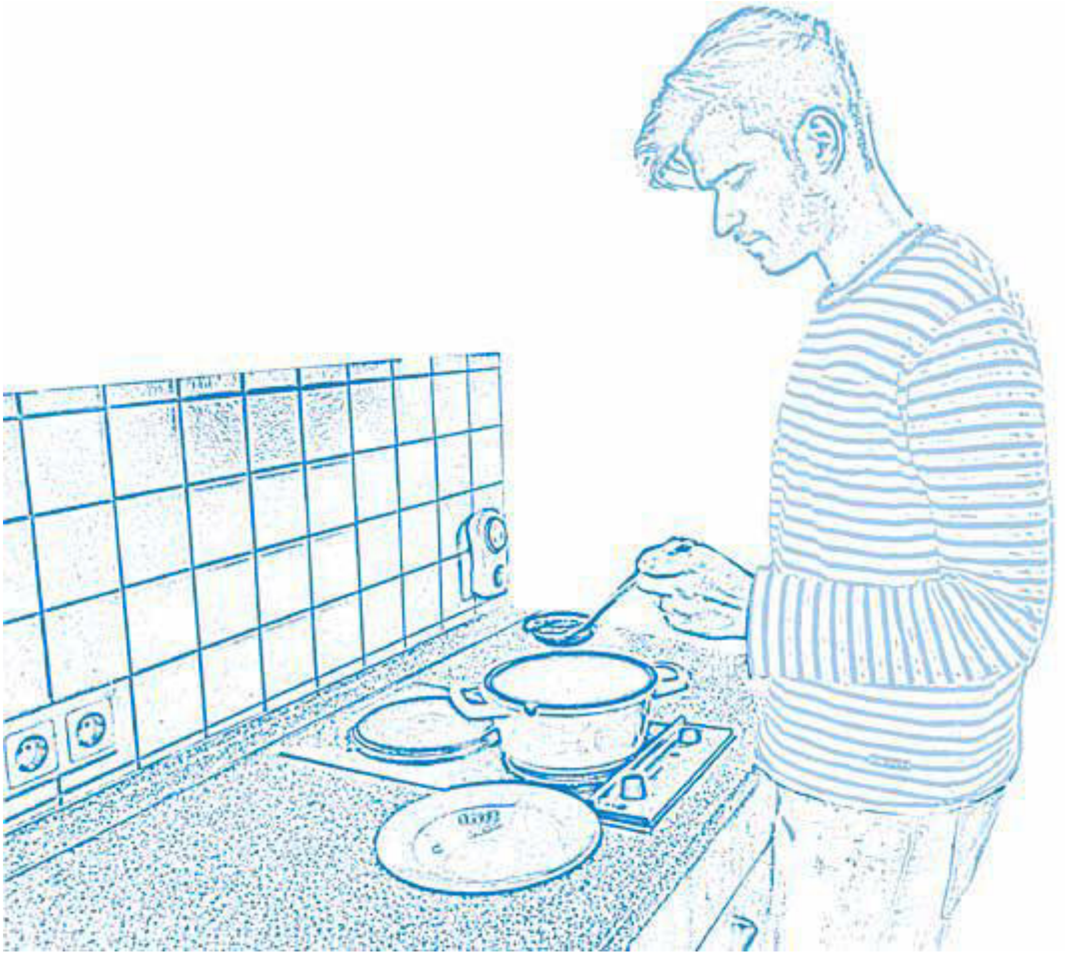

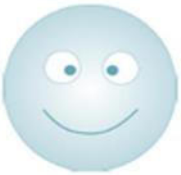

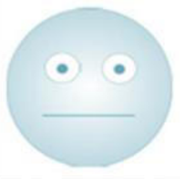

No difficulties **Difficulties**

0------------------------1------------------------2------------------------3

4. Do you have any difficulties to clean a board?

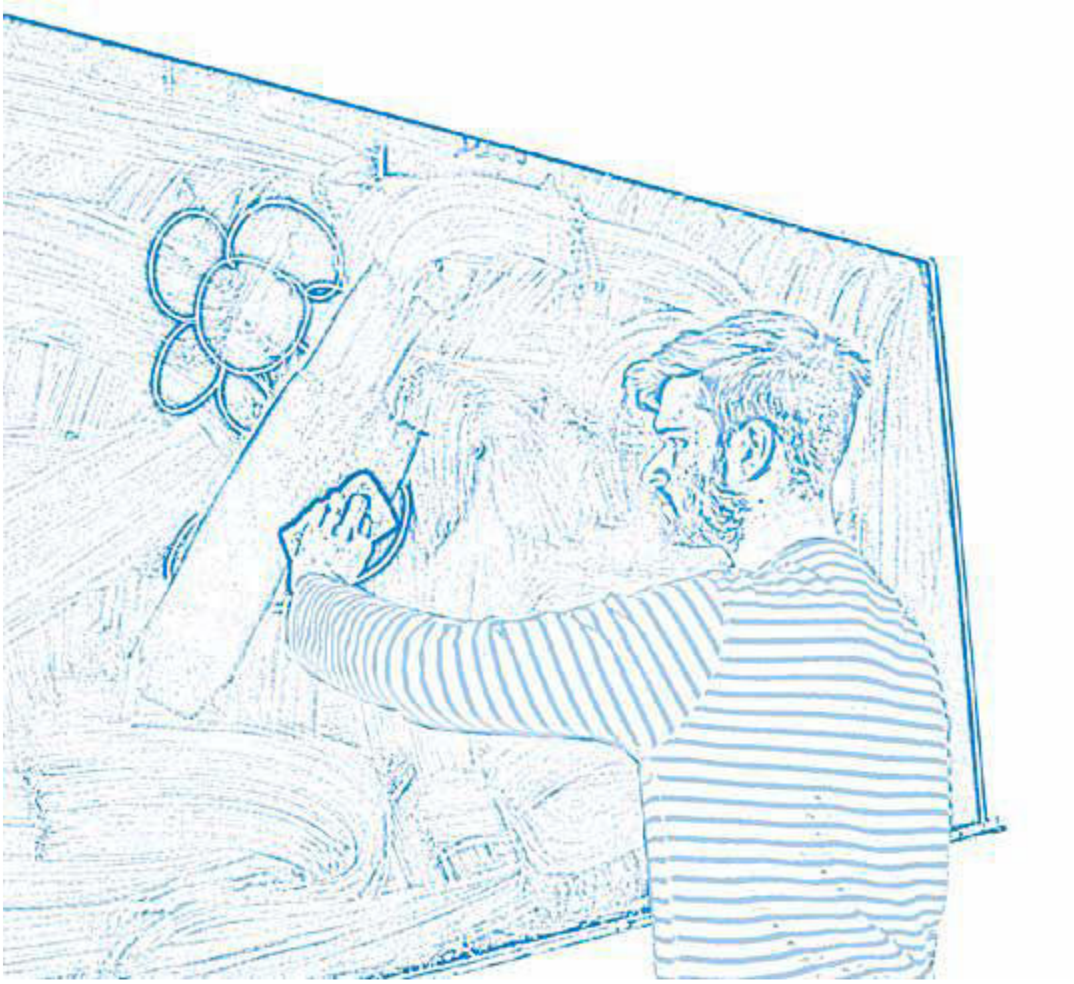

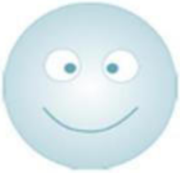

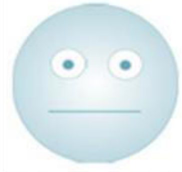

No difficulties **Difficulties**

0-------------------------1-------------------------2------------------------3

5. Do you have any difficulties to tighten a screw?

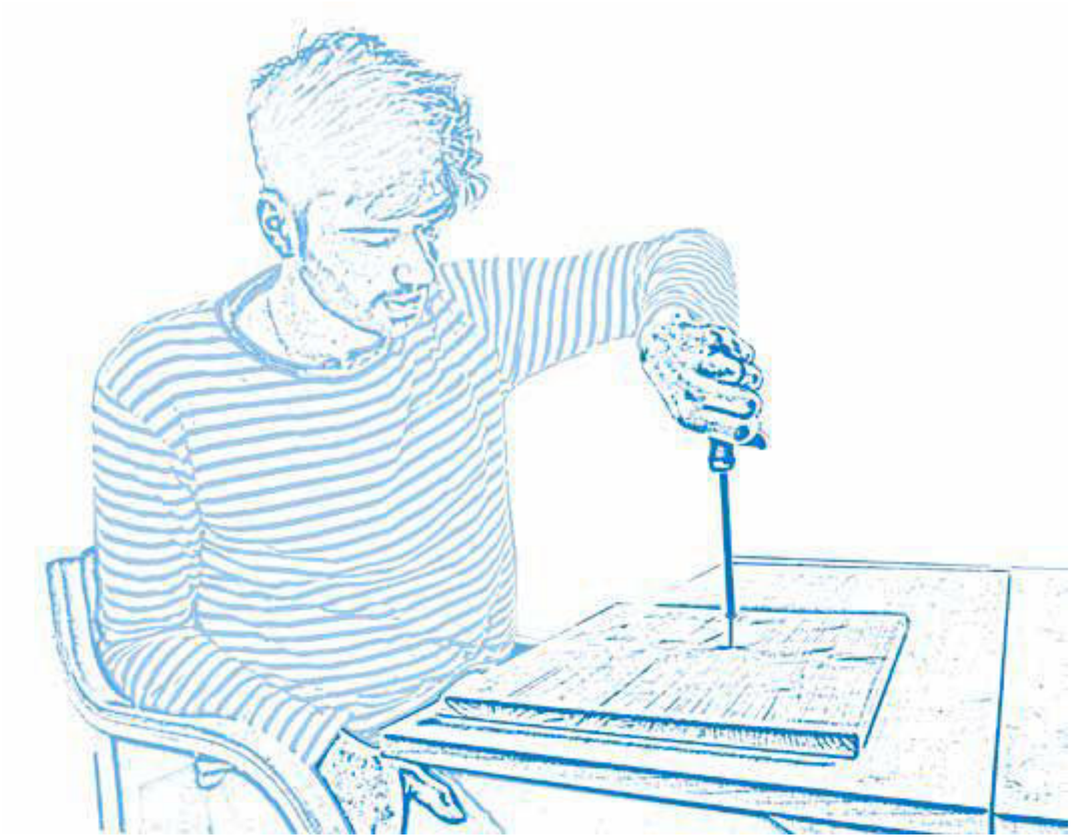

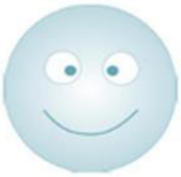

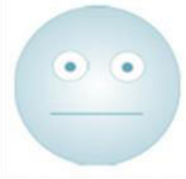

No difficulties **Difficulties**

0------------------------1------------------------2------------------------3

6. Do you have any difficulties to fil a flower pot with potting soil?

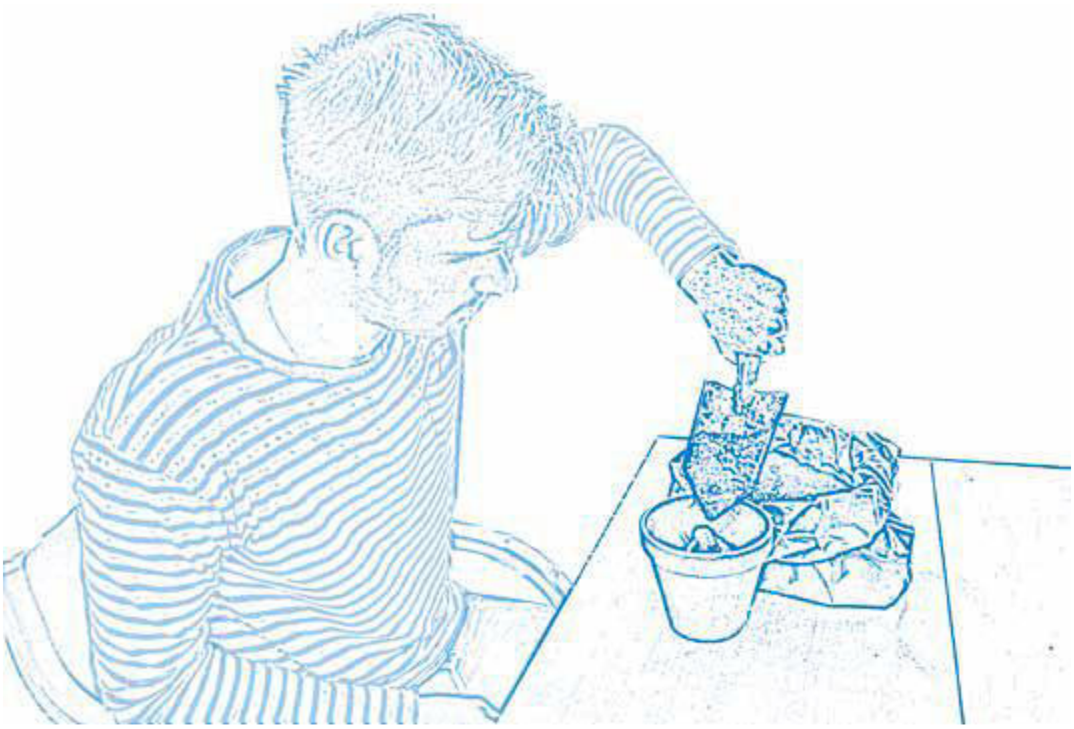

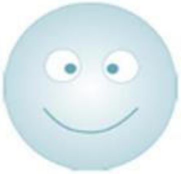

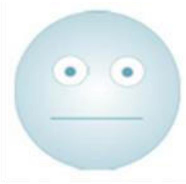

No difficulties **Difficulties**

0------------------------1------------------------2------------------------3

7. **Do you have any difficulties to hang out the laundry?**

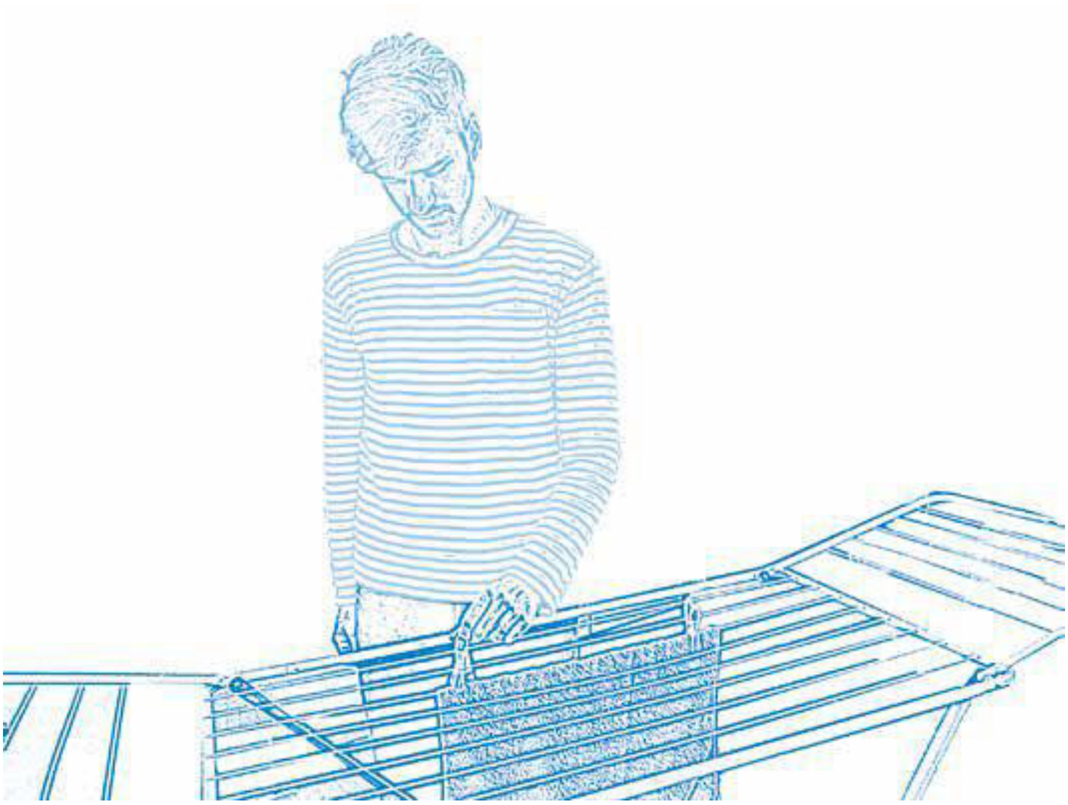

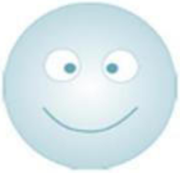

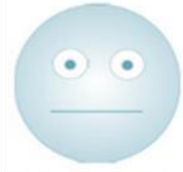

No difficulties **Difficulties**

0------------------------1------------------------2------------------------3

8. Do you have any difficulties to eat soup?

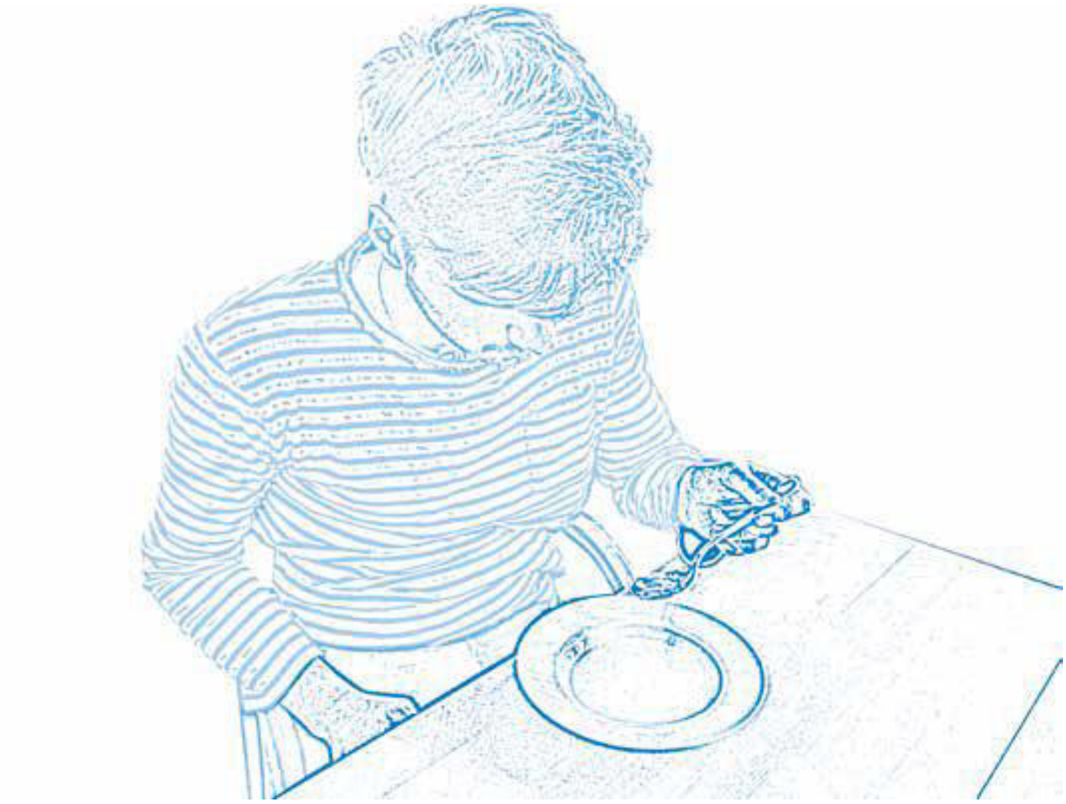

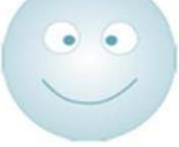

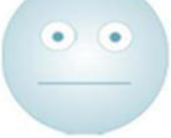

No difficulties **Difficulties**

0------------------------1------------------------2------------------------3

9. Do you have any difficulties to use the phone?

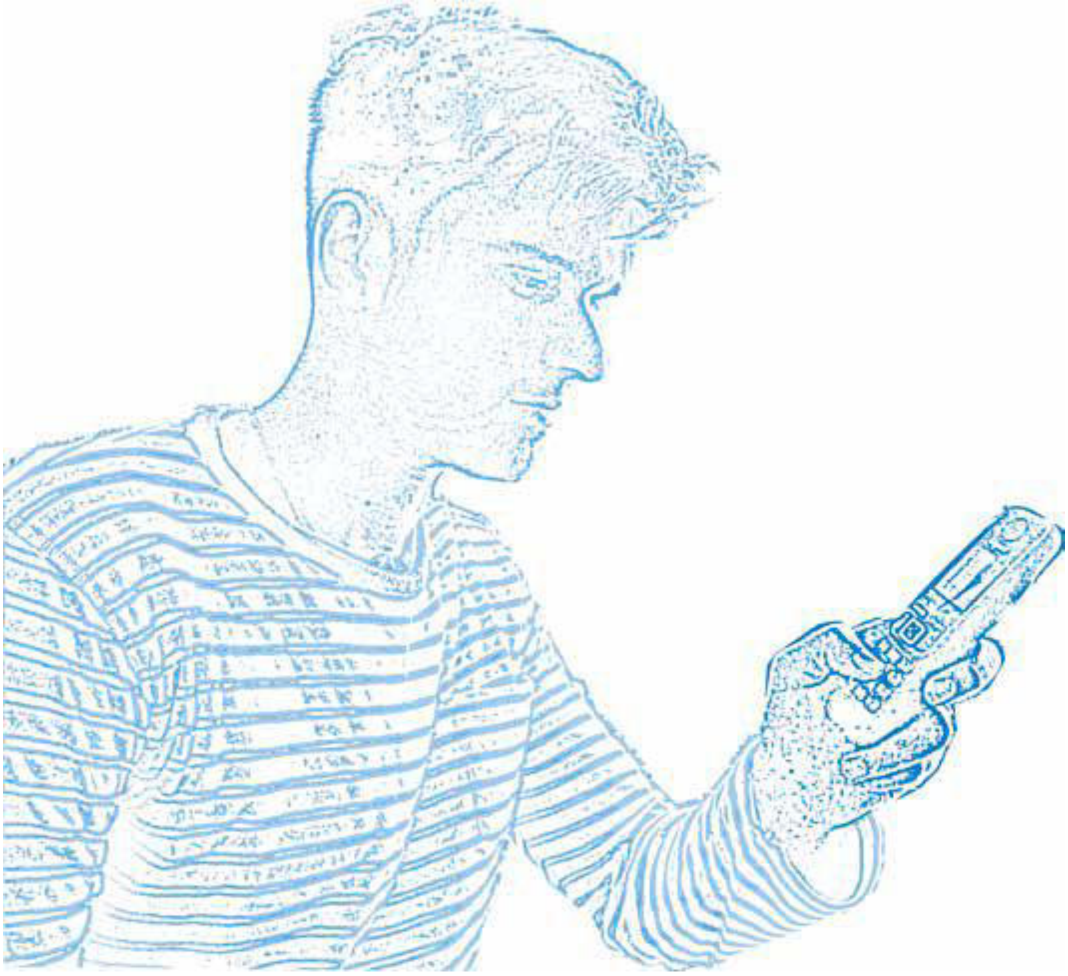

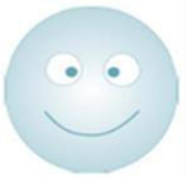

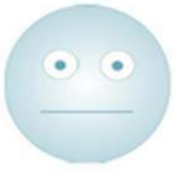

No difficulties **Difficulties**

0------------------------1------------------------2------------------------3

10. Do you have any difficulties to prepare a slice of toast?

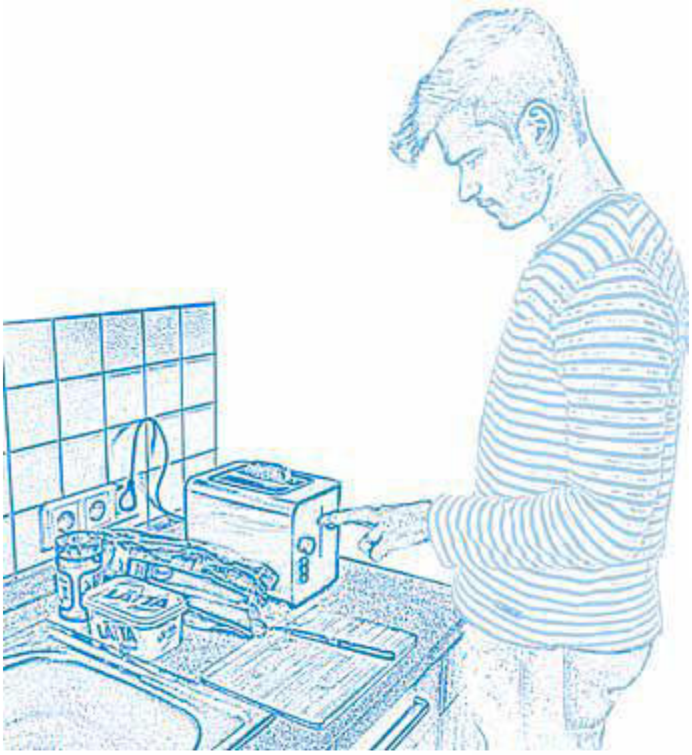

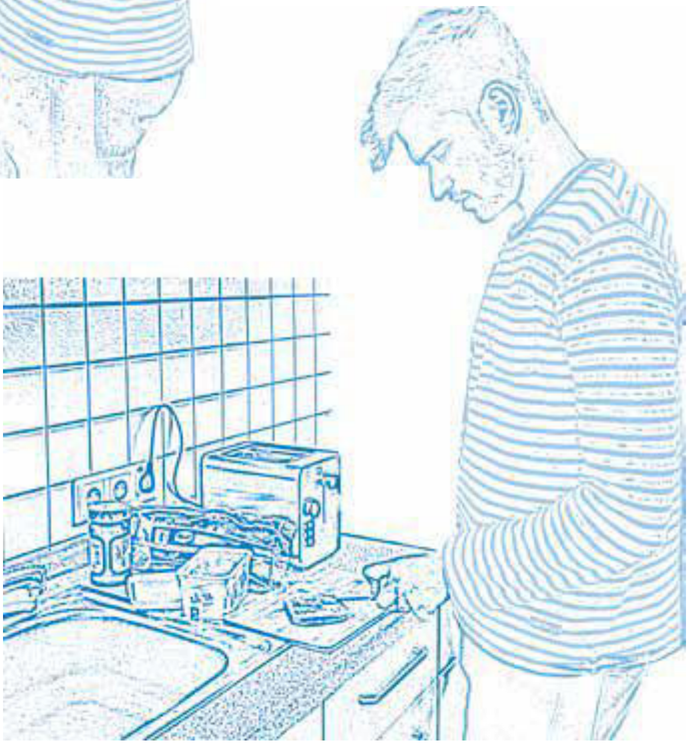

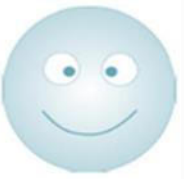

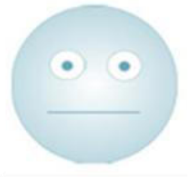

No difficulties **Difficulties**

0------------------------1------------------------2------------------------3

11. Do you have any difficulties to prepare a cup of filter coffee?

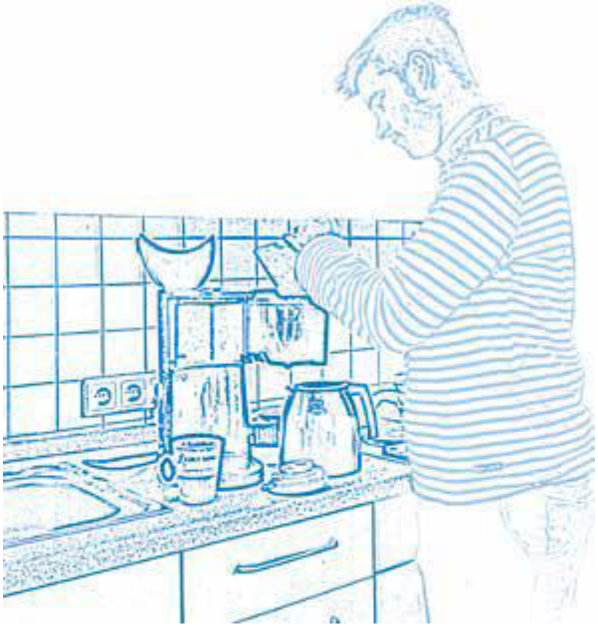

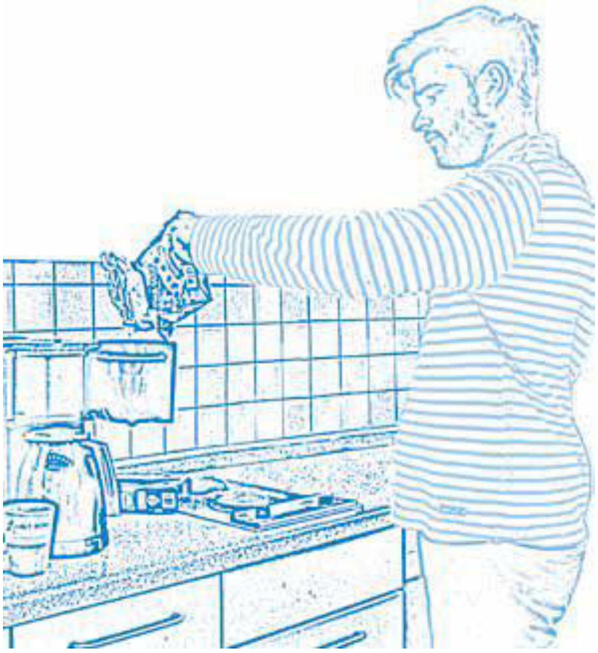

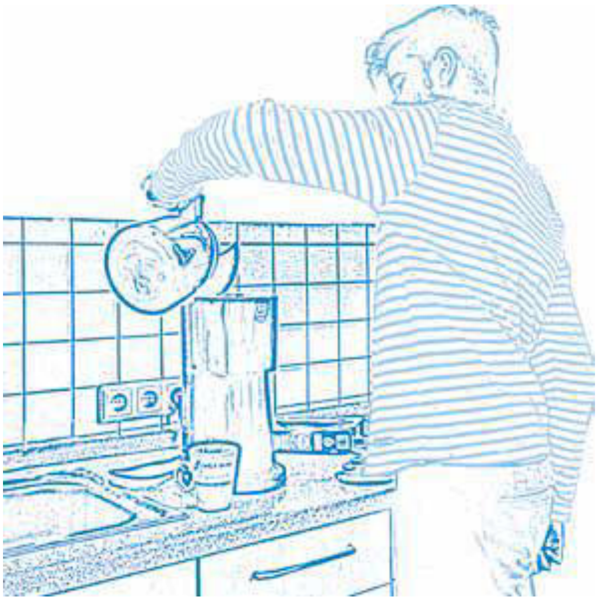

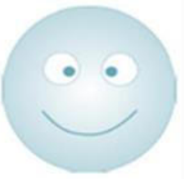

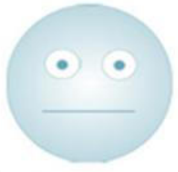

No difficulties **Difficulties**

0-------------------------1-------------------------2-------------------------3

12. Do you have any difficulties to punch a piece of paper and sort it in a folder?

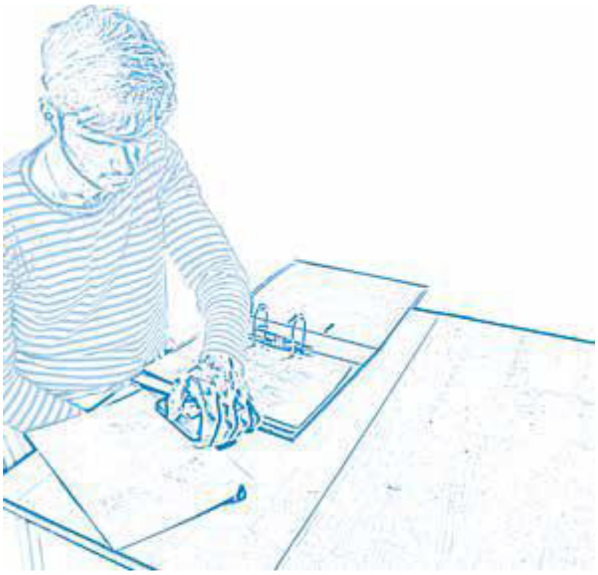

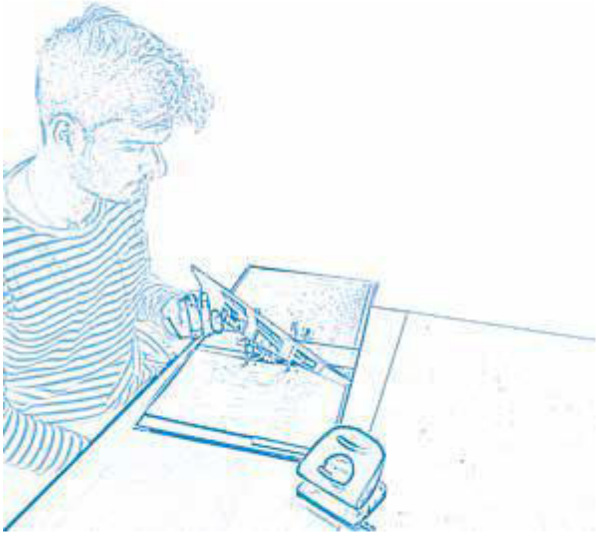

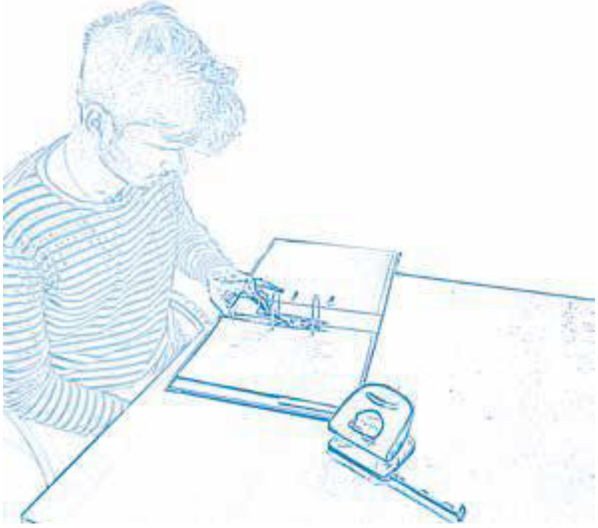

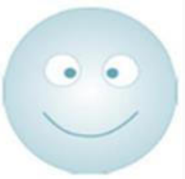

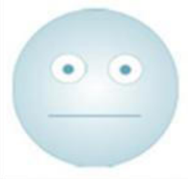

No difficulties **Difficulties**

0------------------------1------------------------2------------------------3

13. Do you have any difficulties to set the table?

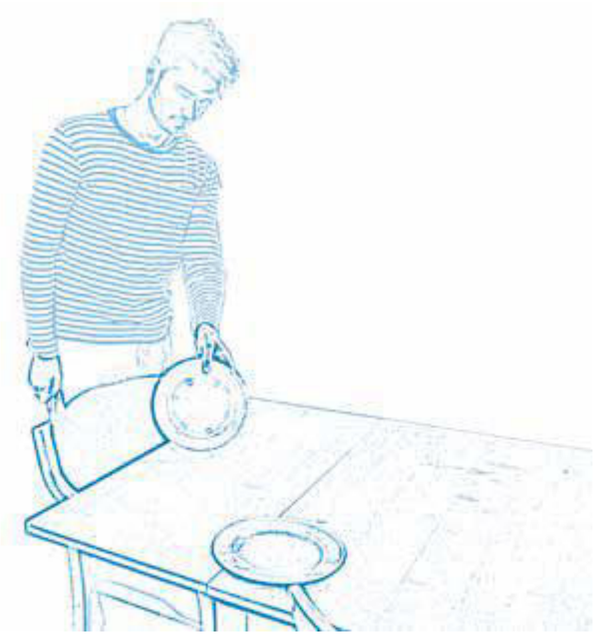

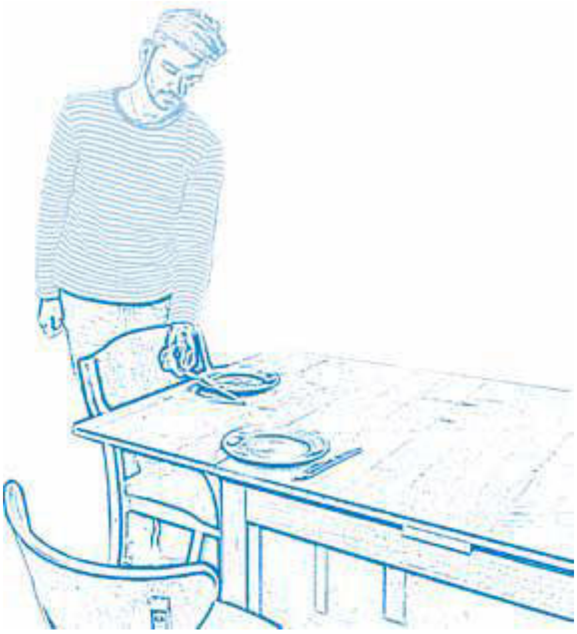

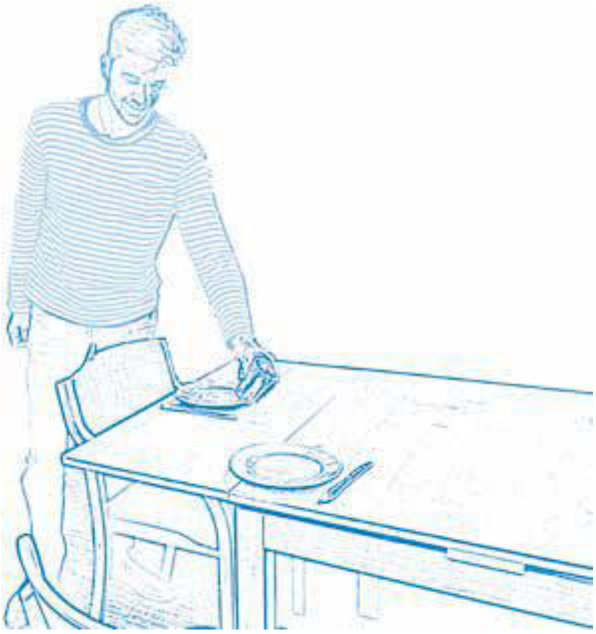

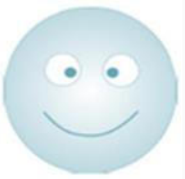

No difficulties

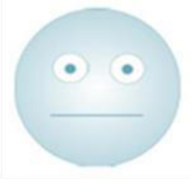

Difficulties

0-------------------------1-------------------------2-------------------------3

14. Do you have any difficulties to jump over a lorry?

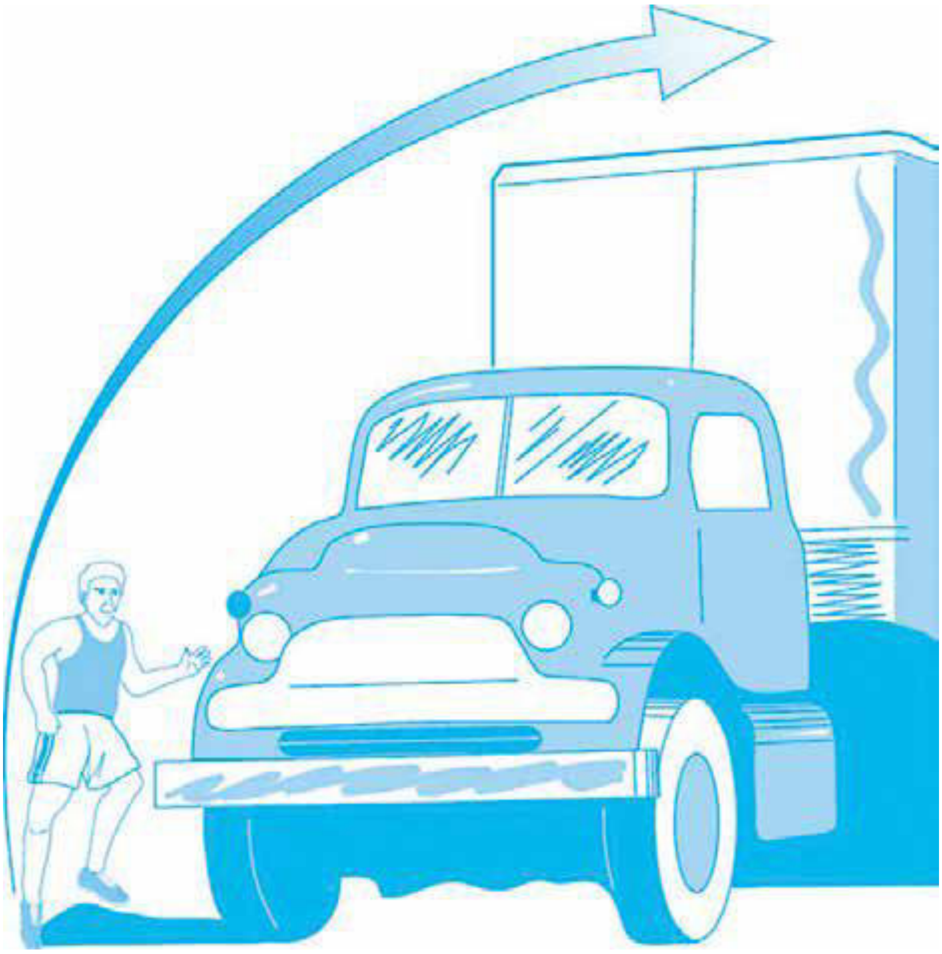

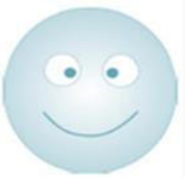

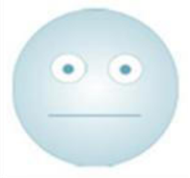

No difficulties **Difficulties**

0------------------------1------------------------2------------------------3

Booklet for the Visual-Analogue Test assessing Anosognosia for Naturalistic Action Tasks

(VATA-NAT)

For Patients with right hemisphere brain damage

VATA-NAT

Instructions

"You now will be asked, how well you **currently** can solve a series of actions. Every task will be shown on a picture. Additionally, the question is written above the picture. I will read out every question to you. Please tell me, how well you can solve the action **at the moment**. The questions are **not** about the mobility of your arm, but generally, how you are able to mentally **plan and execute the actions**.

Below each picture there is an evaluation scale. Please show me your capacity on this scale ranging from 0 to 3. Zero means, that you have no problem at all to solve the requested action at this moment. One means minor difficulties and two means serious difficulties. Three means, that it is currently not possible for you to solve the requested action. You can show me your answer by pointing on the appropriate position on the scale. Let´s start with a practice item."

Example: Do you have any difficulties to drive a car?

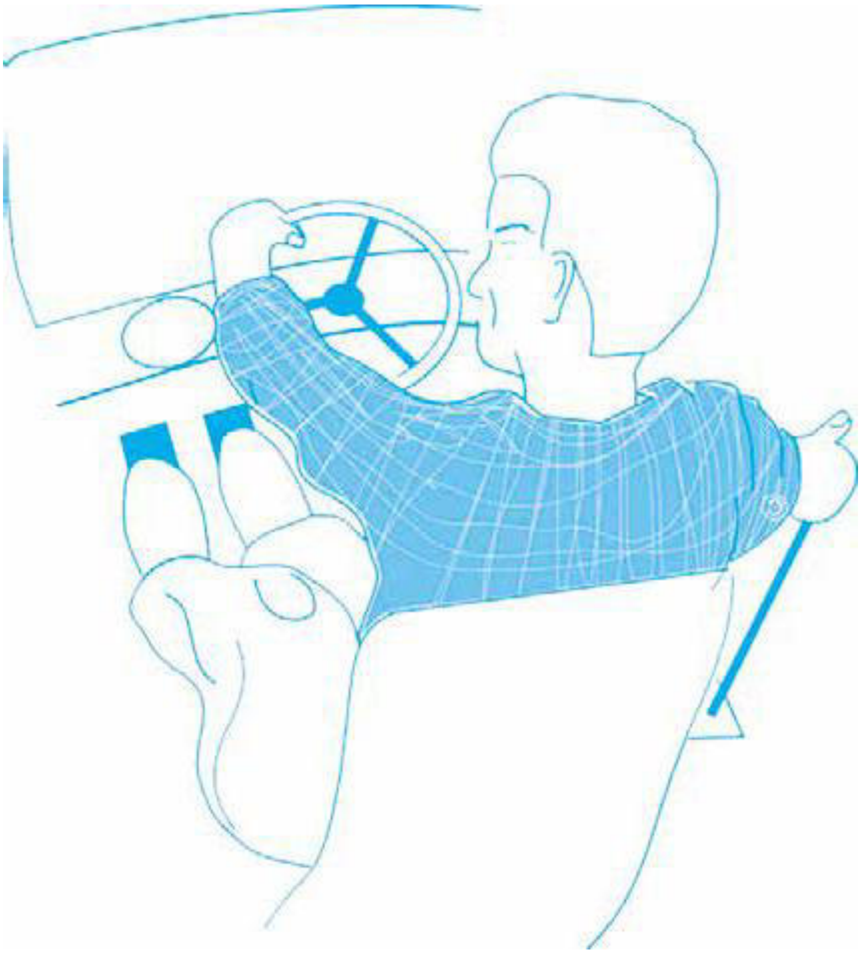

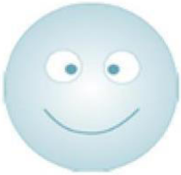

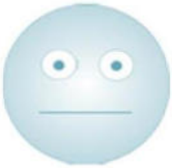

No difficulties **Difficulties**

0-------------------------1-------------------------2-------------------------3

1. Do you have any difficulties to take a fried egg out of a pan?

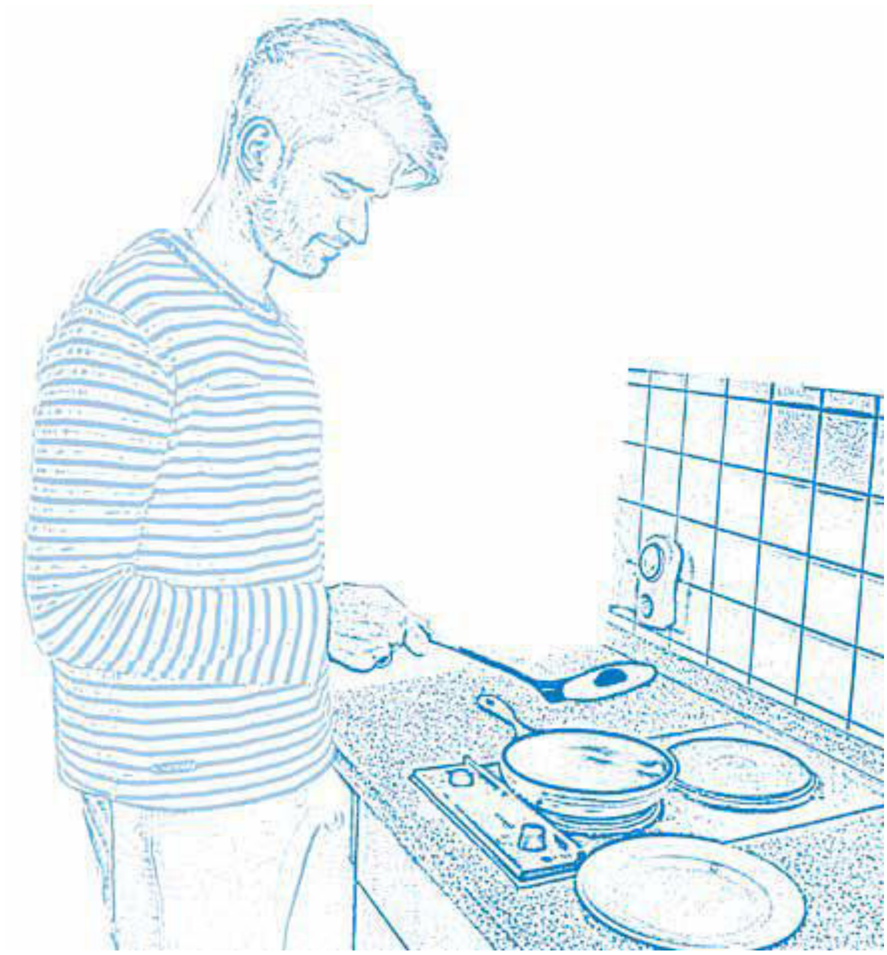

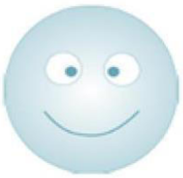

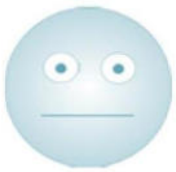

No difficulties **Difficulties**

0------------------------1------------------------2------------------------3

2. Do you have any difficulties to open a bottle with crown cap?

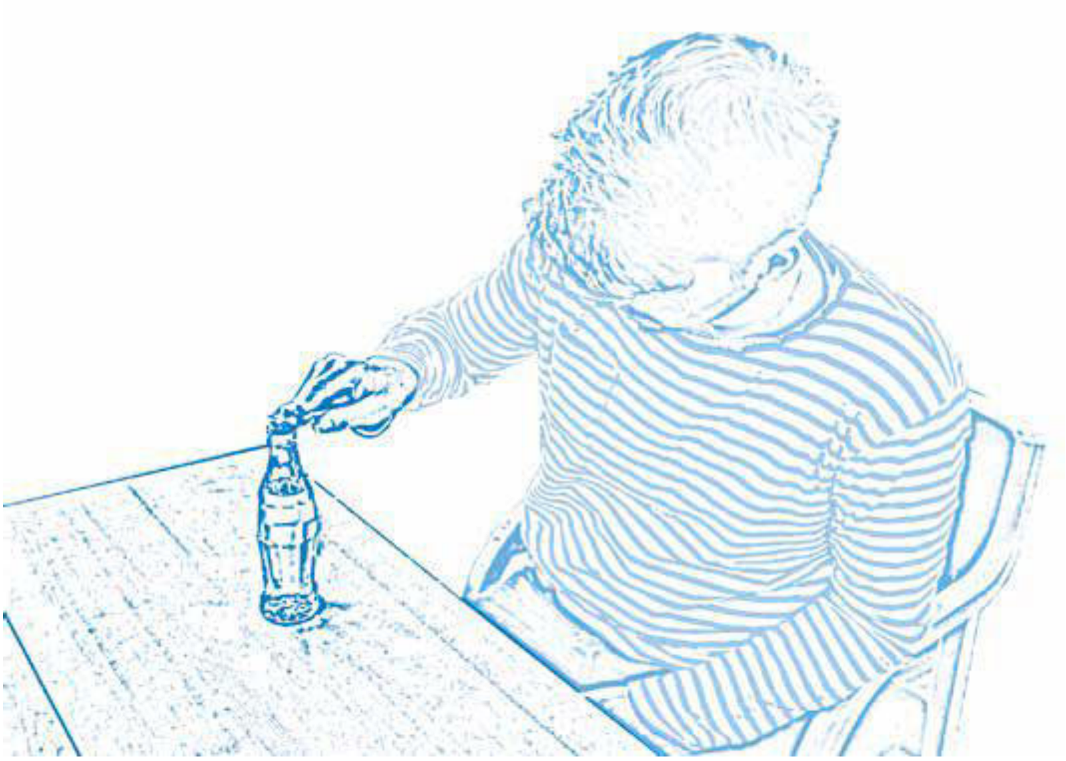

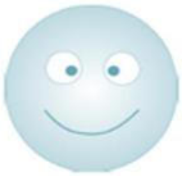

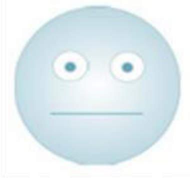

No difficulties **Difficulties**

0-----------------------1-----------------------2-----------------------3

3. Do you have any difficulties to scoop soup out of a pot?

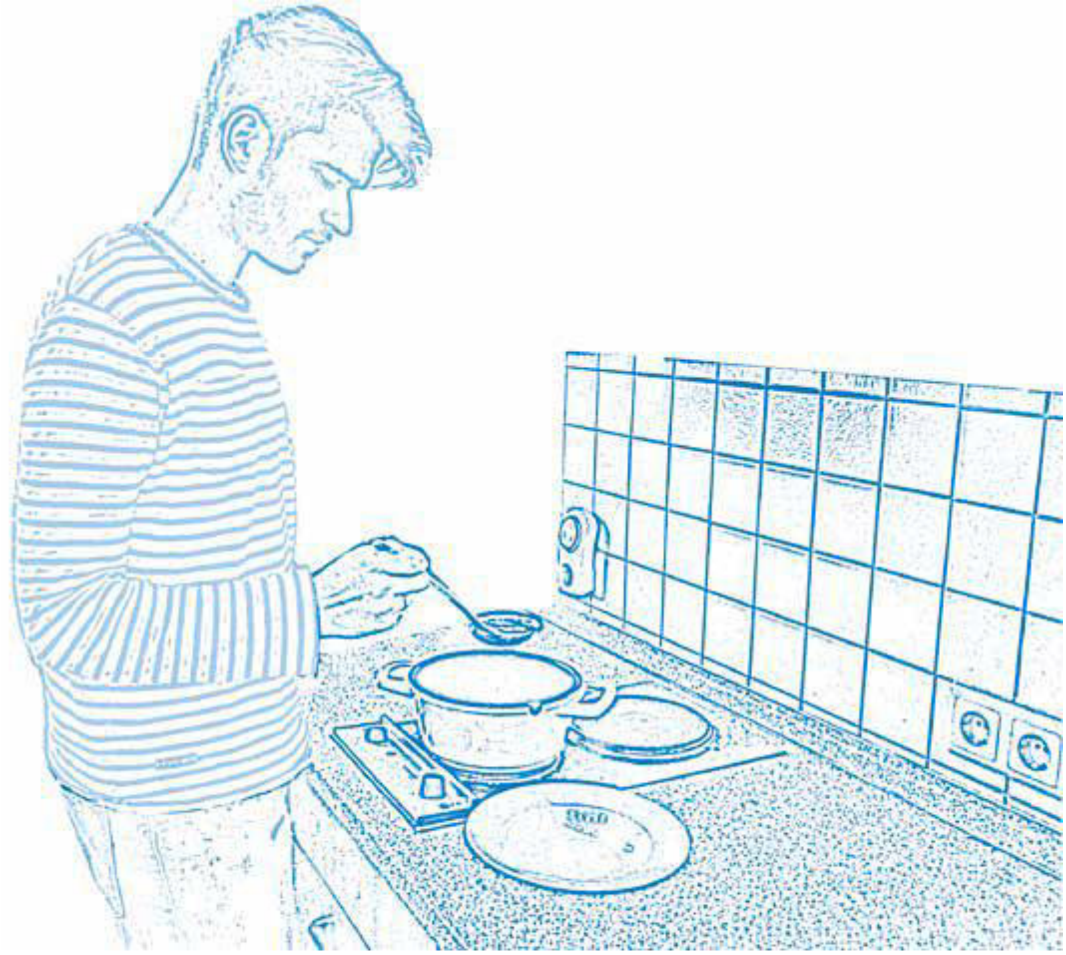

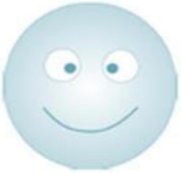

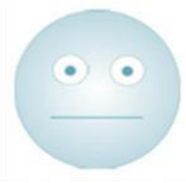

No difficulties **Difficulties**

0------------------------1------------------------2------------------------3

4. Do you have any difficulties to clean a board?

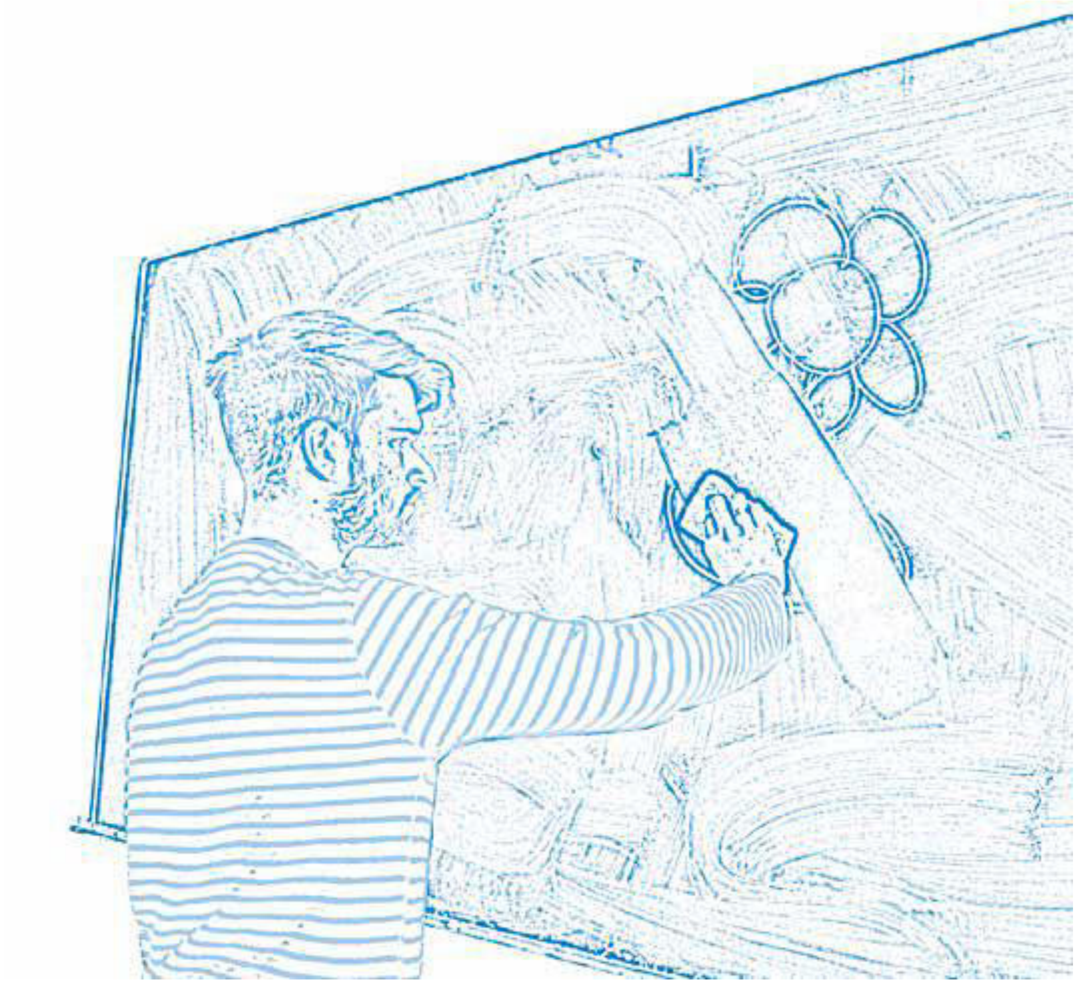

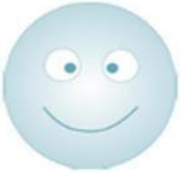

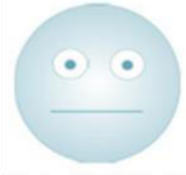

No difficulties **Difficulties**

0-------------------------1-------------------------2------------------------3

5. Do you have any difficulties to tighten a screw?

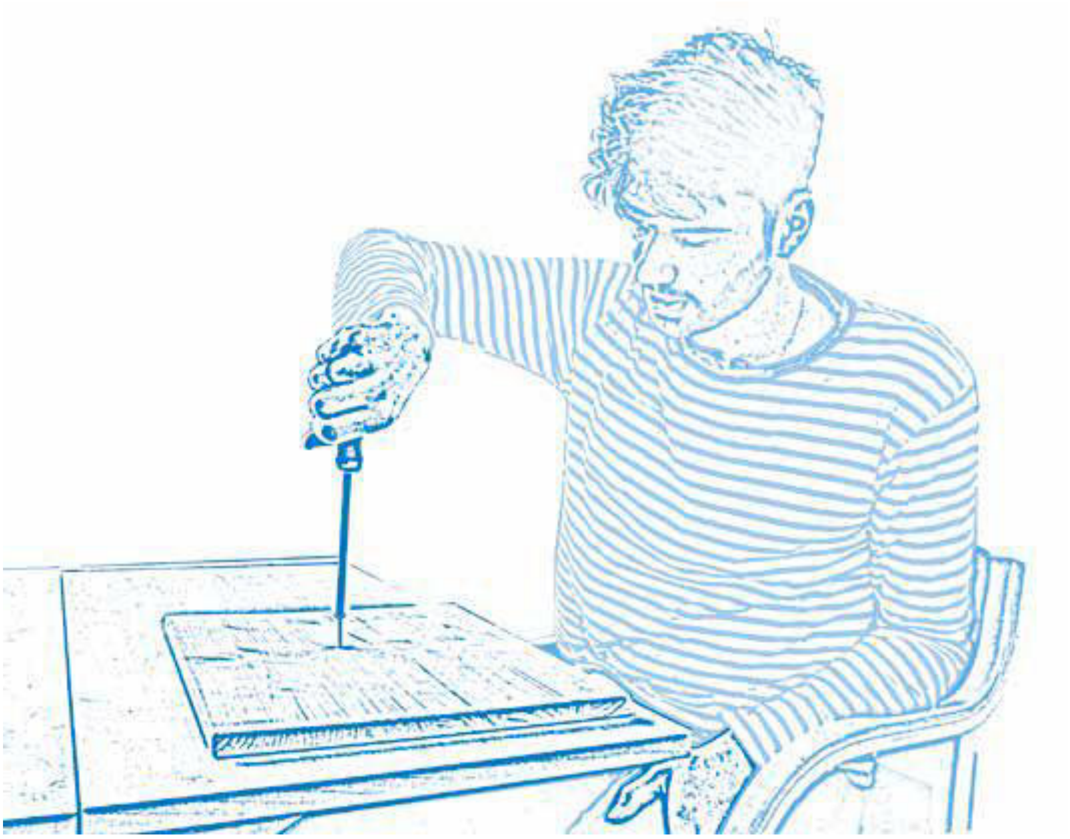

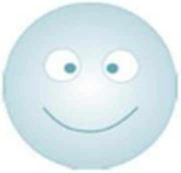

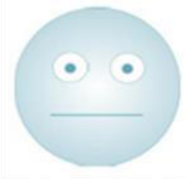

No difficulties **Difficulties**

0------------------------1------------------------2------------------------3

6. **Do you have any difficulties to fil a flower pot with potting soil?**

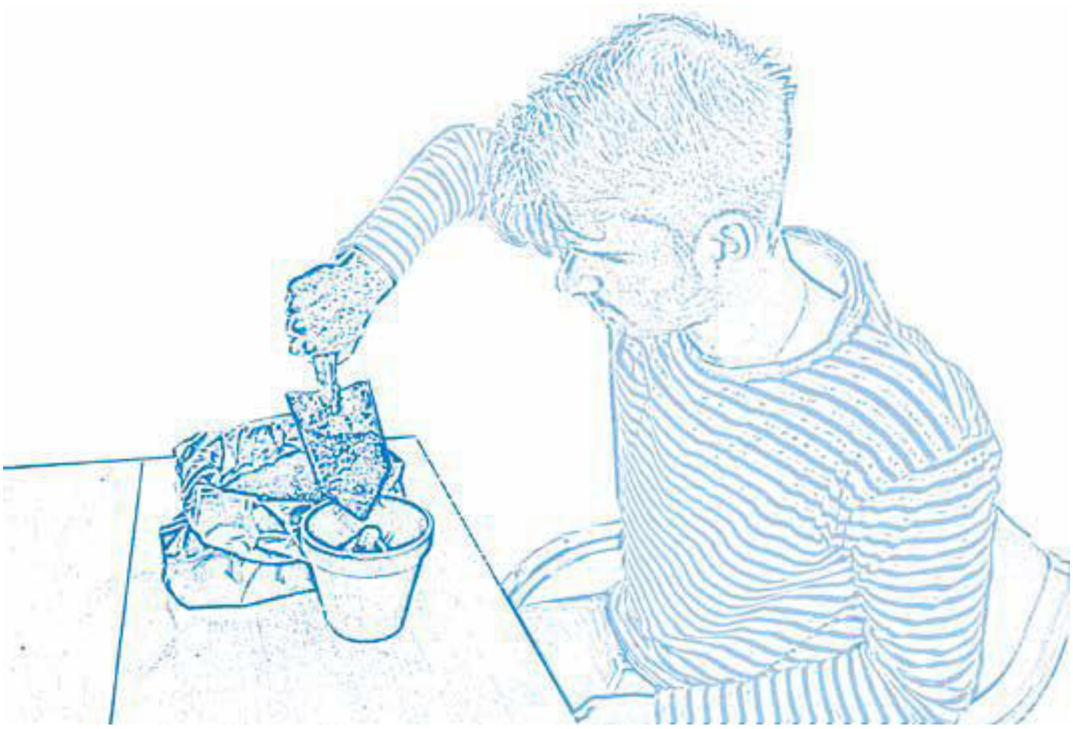

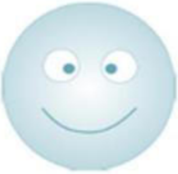

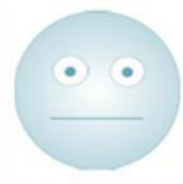

No difficulties **Difficulties**

0------------------------1------------------------2------------------------3

7. Do you have any difficulties to hang out the laundry?

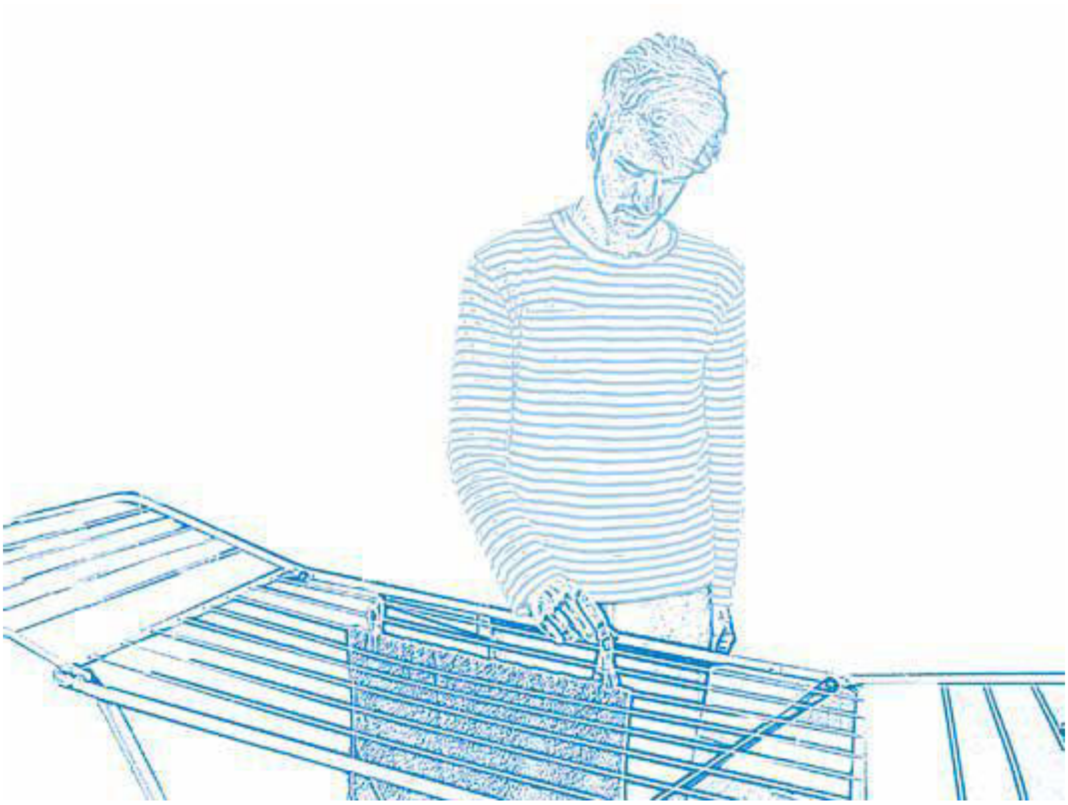

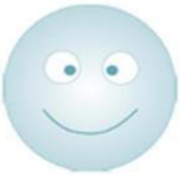

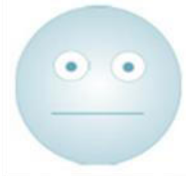

No difficulties **Difficulties**

0------------------------1------------------------2------------------------3

8. Do you have any difficulties to eat soup?

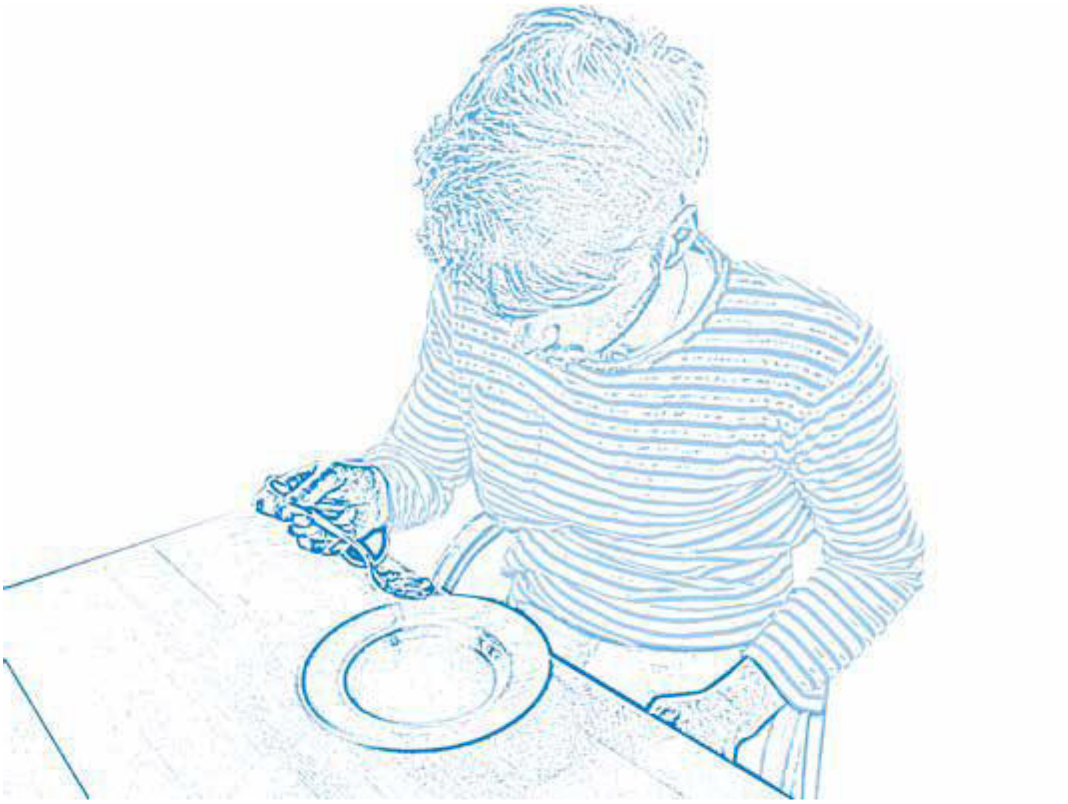

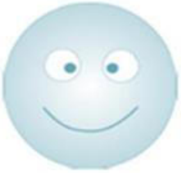

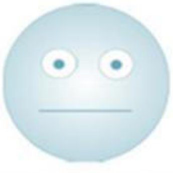

No difficulties **Difficulties**

0-------------------------1------------------------2------------------------3

9. Do you have any difficulties to use the phone?

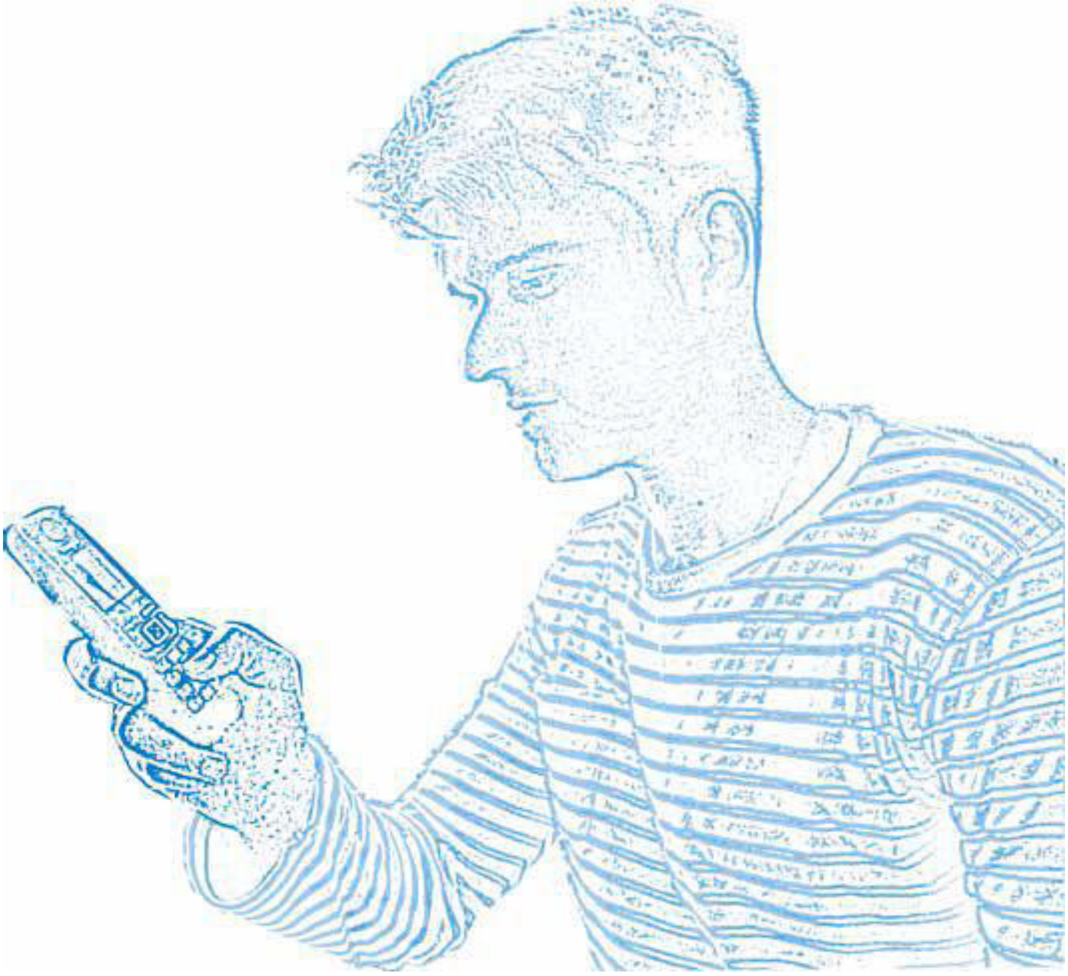

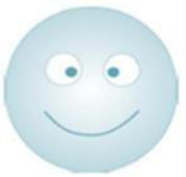

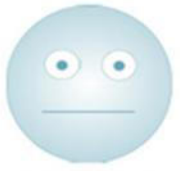

No difficulties **Difficulties**

0------------------------1------------------------2------------------------3

10. Do you have any difficulties to prepare a slice of toast?

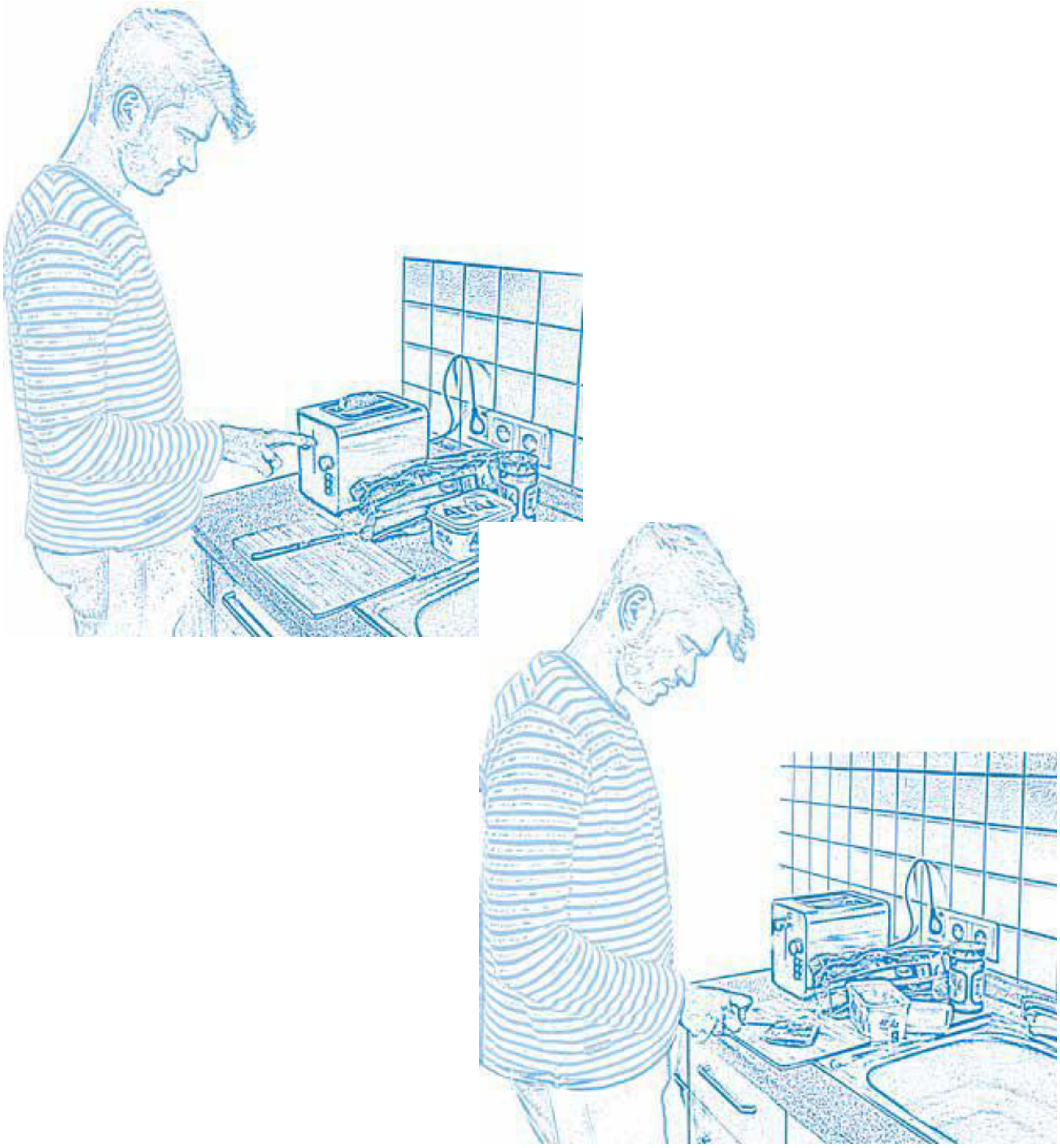

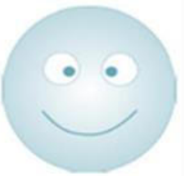

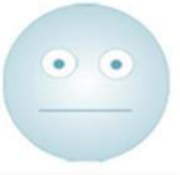

No difficulties **Difficulties**

0------------------------1------------------------2------------------------3

11. Do you have any difficulties to prepare a cup of filter coffee?

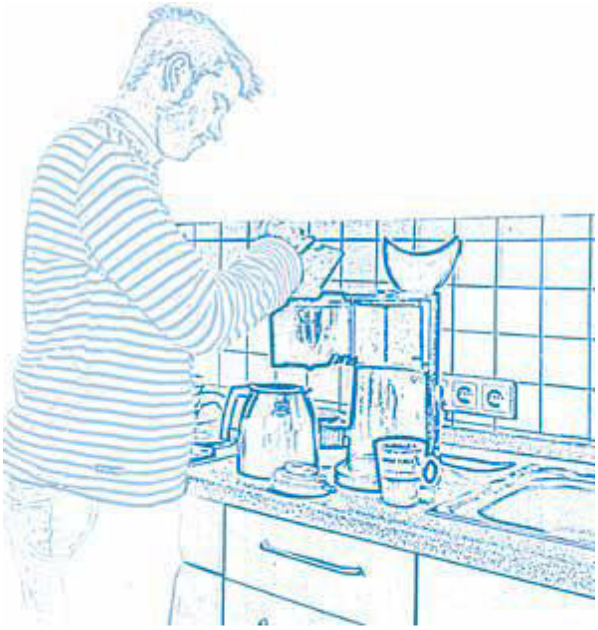

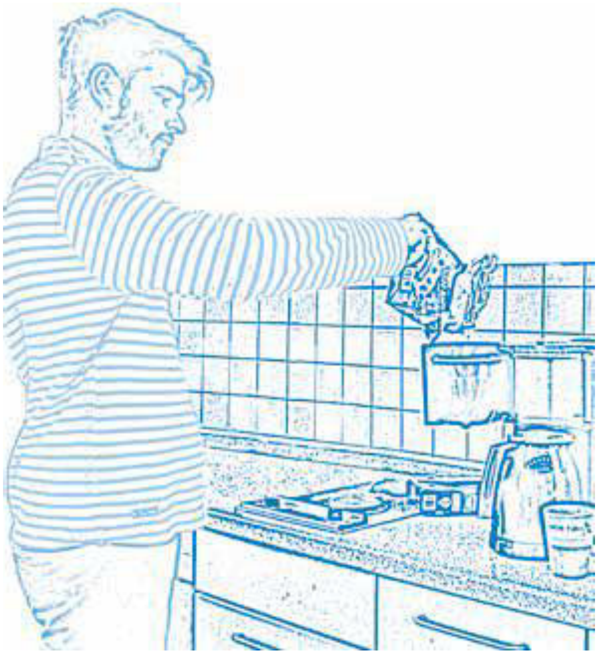

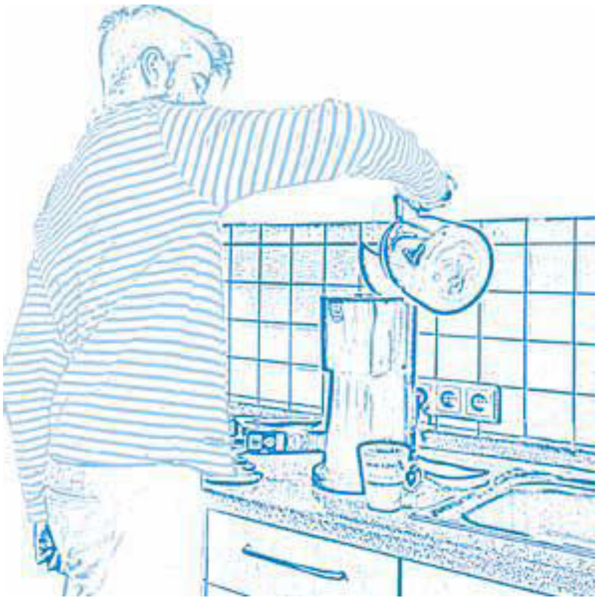

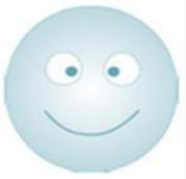

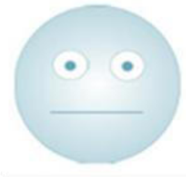

No difficulties **Difficulties**

0------------------------1------------------------2------------------------3

12. Do you have any difficulties to punch a piece of paper and sort it in a folder?

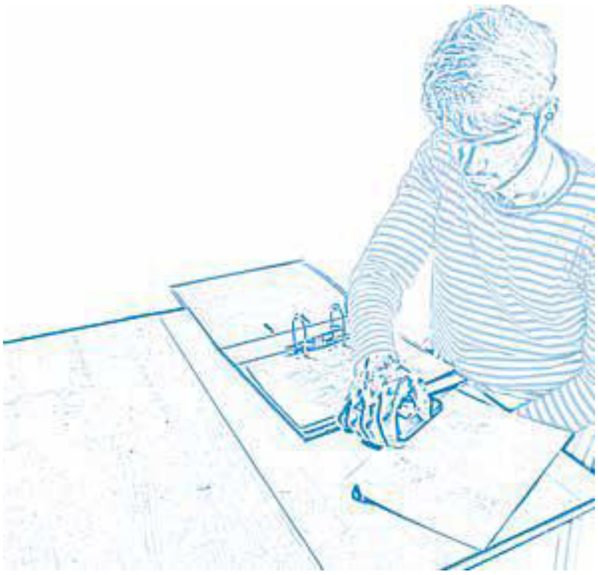

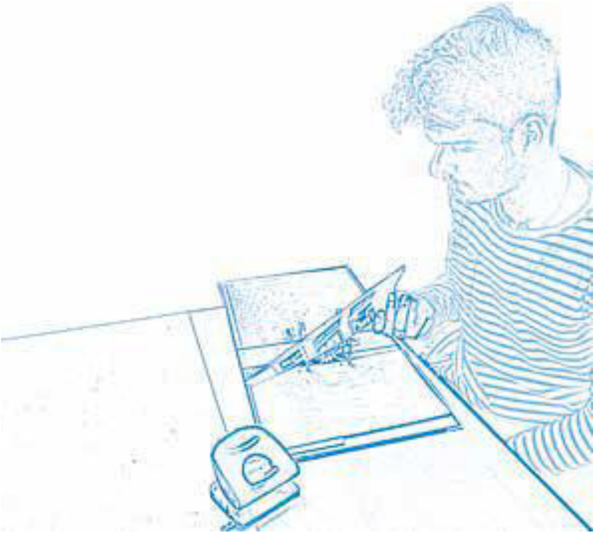

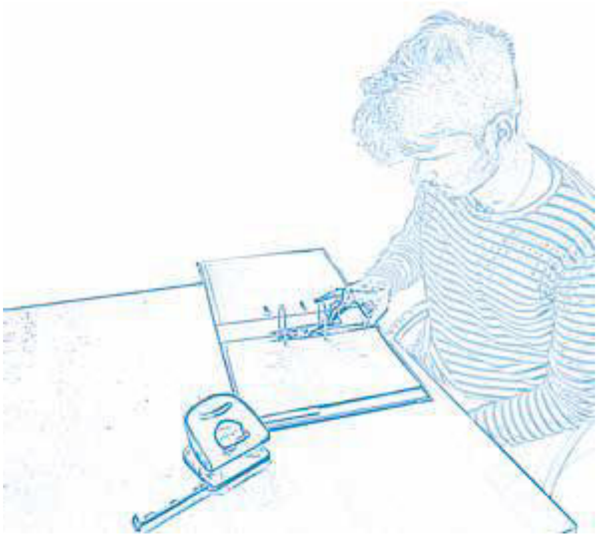

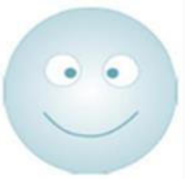

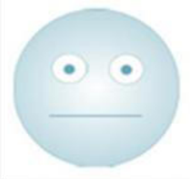

No difficulties **Difficulties**

0------------------------1------------------------2------------------------3

13. Do you have any difficulties to set the table?

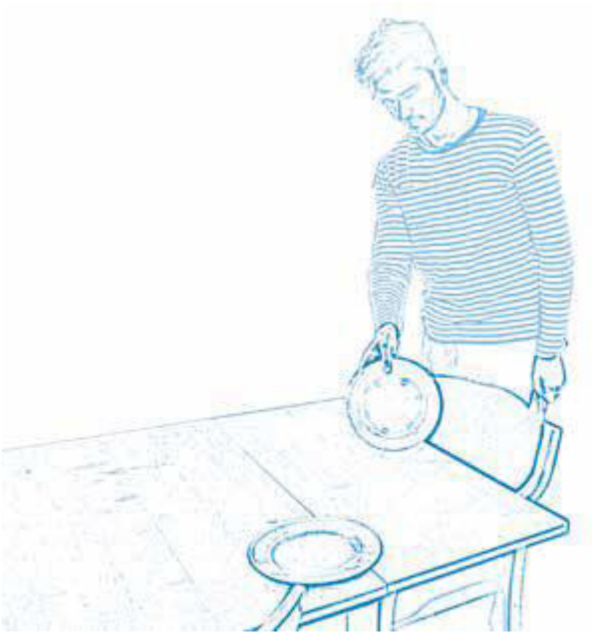

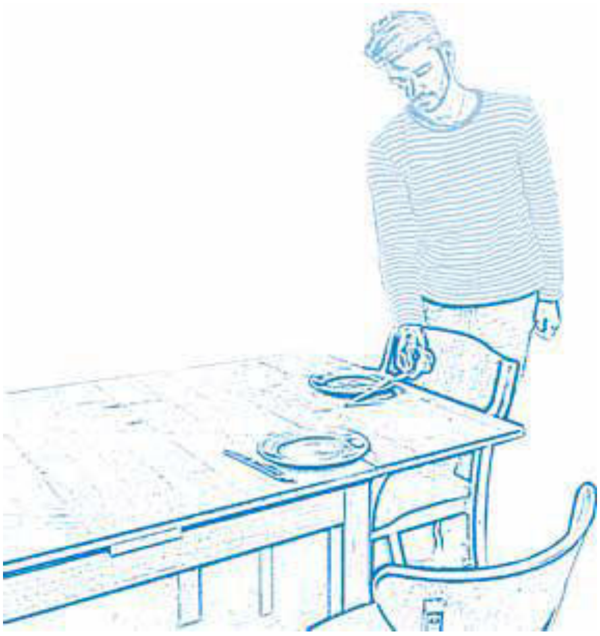

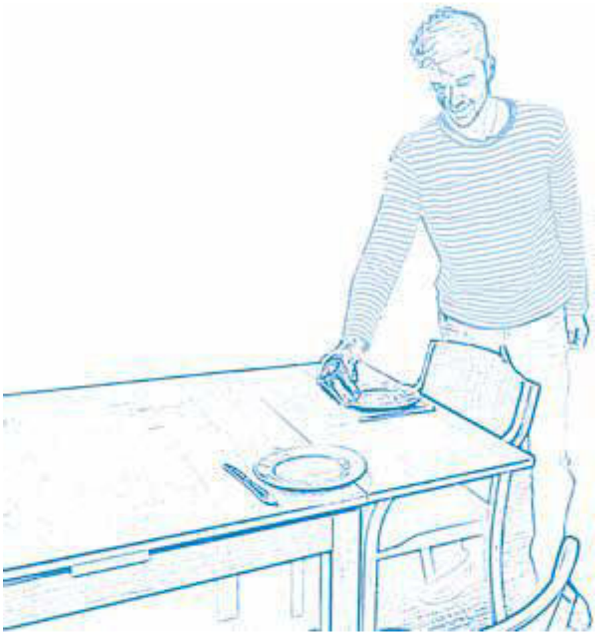

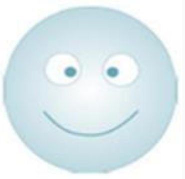

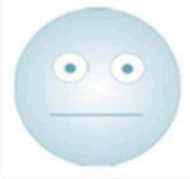

No difficulties **Difficulties**

0-------------------------1-------------------------2-------------------------3

14. Do you have any difficulties to jump over a lorry?

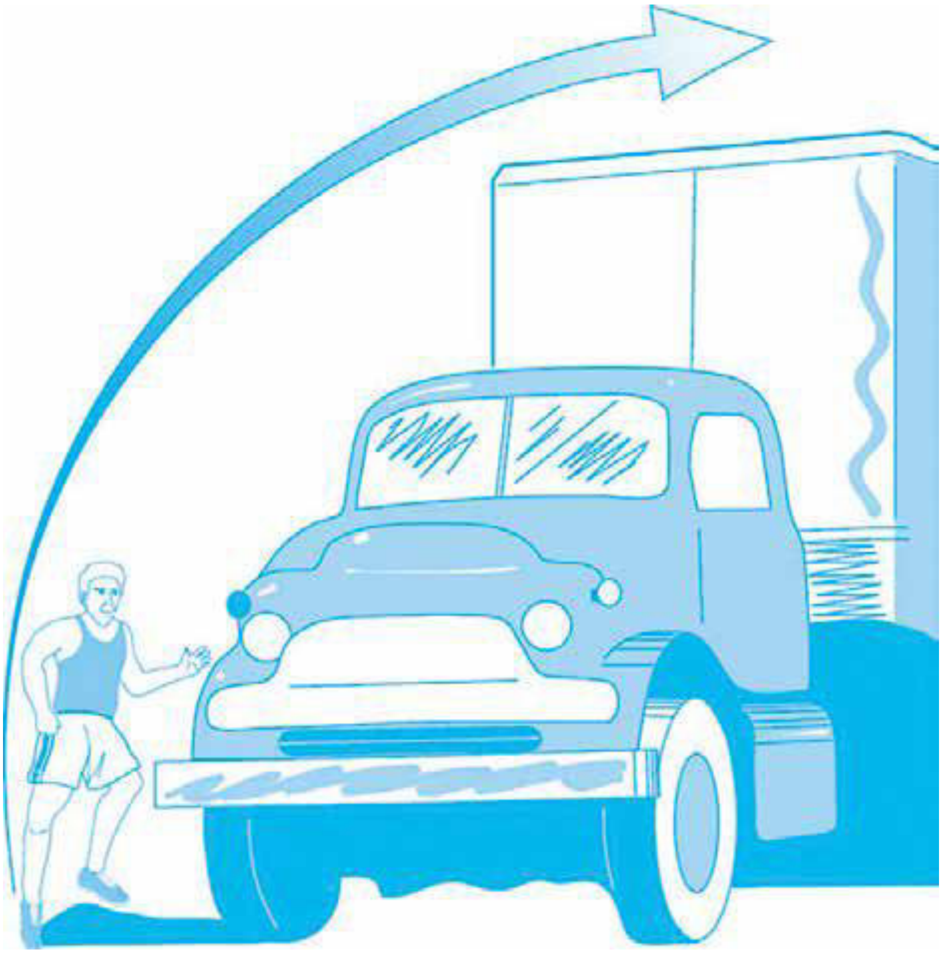

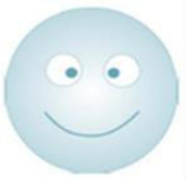

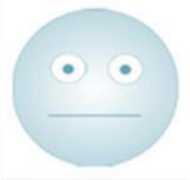

No difficulties **Difficulties**

0------------------------1------------------------2------------------------3
